# Structural Dynamics of C_2_F_4_I_2_ in Cyclohexane Studied via Time-Resolved X-ray Liquidography

**DOI:** 10.3390/ijms22189793

**Published:** 2021-09-10

**Authors:** Jain Gu, Seonggon Lee, Seunghwan Eom, Hosung Ki, Eun Hyuk Choi, Yunbeom Lee, Shunsuke Nozawa, Shin-ichi Adachi, Jeongho Kim, Hyotcherl Ihee

**Affiliations:** 1 Department of Chemistry and KI for the BioCentury, Korea Advanced Institute of Science and Technology (KAIST), Daejeon 34141, Korea; gujain33@kaist.ac.kr (J.G.); infinite_l@kaist.ac.kr (S.L.); eesshh@kaist.ac.kr (S.E.); kihosung@kaist.ac.kr (H.K.); horang2@kaist.ac.kr (E.H.C.); ask123@kaist.ac.kr (Y.L.); 2 Center for Nanomaterials and Chemical Reactions, Institute for Basic Science (IBS), Daejeon 34141, Korea; 3 Institute of Materials Structure Science, High Energy Accelerator Research Organization, 1-1 Oho, Tsukuba 305-0801, Ibaraki, Japan; noz@post.kek.jp (S.N.); shinichi.adachi@kek.jp (S.-i.A.); 4 Department of Materials Structure Science, School of High Energy Accelerator Science, The Graduate University for Advanced Studies, 1-1 Oho, Tsukuba 305-0801, Ibaraki, Japan; 5 Department of Chemistry, Inha University, 100 Inha-ro, Michuhol-gu, Incheon 22212, Korea; jkim5@inha.ac.kr

**Keywords:** time-resolved X-ray liquidography, 1,2-diiodotetrafluoroethane, haloalkane, structural dynamics, photodissociation, isomer, stereochemistry

## Abstract

The halogen elimination of 1,2-diiodoethane (C_2_H_4_I_2_) and 1,2-diiodotetrafluoroethane (C_2_F_4_I_2_) serves as a model reaction for investigating the influence of fluorination on reaction dynamics and solute–solvent interactions in solution-phase reactions. While the kinetics and reaction pathways of the halogen elimination reaction of C_2_H_4_I_2_ were reported to vary substantially depending on the solvent, the solvent effects on the photodissociation of C_2_F_4_I_2_ remain to be explored, as its reaction dynamics have only been studied in methanol. Here, to investigate the solvent dependence, we conducted a time-resolved X-ray liquidography (TRXL) experiment on C_2_F_4_I_2_ in cyclohexane. The data revealed that (ⅰ) the solvent dependence of the photoreaction of C_2_F_4_I_2_ is not as strong as that observed for C_2_H_4_I_2_, and (ⅱ) the nongeminate recombination leading to the formation of I_2_ is slower in cyclohexane than in methanol. We also show that the molecular structures of the relevant species determined from the structural analysis of TRXL data provide an excellent benchmark for DFT calculations, especially for investigating the relevance of exchange-correlation functionals used for the structural optimization of haloalkanes. This study demonstrates that TRXL is a powerful technique to study solvent dependence in the solution phase.

## 1. Introduction

The photoexcitation of haloalkanes at ultraviolet wavelengths induces an electronic transition corresponding to the n → σ* transition of a carbon–halogen bond, leading to the dissociation of halogen atoms [1,2]. This photoinduced dissociation has served as an excellent model system to study reaction dynamics due to their simple molecular structures [1,2,3,4,5,6,7,8,9,10,11,12,13,14,15,16,17,18,19,20,21,22,23,24,25,26,27,28,29,30,31,32,33,34,35,36,37,38,39,40,41,42,43,44,45,46,47,48]. The structures of reaction intermediates of haloalkane photodissociation are relevant to the stereochemical control of the products of the halogen elimination reaction of haloalkanes. For example, according to the Skell hypothesis [49,50], it was proposed that a *bridged* radical, where the rotation of its C−C bond is prohibited, is key to the observed stereochemical control of the products. In addition, haloalkanes serve as a good model system for studying the effect of atomic substitution on reaction dynamics. For example, fluorination, which is the replacement of hydrogen with fluorine, often alters the chemical properties and reactivities of molecules. In this regard, photoreactions of 1,2-diiodoethane (C_2_H_4_I_2_) and its fluorinated analog, 1,2-diiodotetrafluoroethane (C_2_F_4_I_2_), were extensively studied as prototypes to explore the effect of fluorination on reaction dynamics and the molecular structures of reaction intermediates [9,10,11,12].

To investigate the reaction dynamics and structures of reaction intermediates, and subtle effects of atomic substitution, the molecular structures of reacting molecules must be characterized. In this regard, time-resolved X-ray liquidography (TRXL), also known as time-resolved X-ray solution scattering, is an excellent technique because it can be used to both provide global reaction pathways of chemical reactions and reveal the structures of transient intermediates in the liquid solution phase [5,6,7,8,9,10,11,12,13,14,15,16,51,52,53,54,55,56,57,58,59,60,61,62,63,64,65,66,67,68,69,70,71,72,73,74,75]. Thus far, TRXL has been used for studying the structural dynamics of a wide variety of systems spanning diatomic molecules, organometallic complexes, proteins, and nanoparticles in the solution phase [5,6,7,8,9,10,11,12,13,14,15,16,51,52,53,54,55,56,57,58,59,60,61,62,63,64,65,66,67,68,69,70,71,72,73,74,75].

In fact, the photodissociation dynamics of both C_2_H_4_I_2_ and C_2_F_4_I_2_ were previously investigated with TRXL. Those TRXL studies on C_2_H_4_I_2_ and C_2_F_4_I_2_ in methanol showed that C_2_F_4_I· and C_2_H_4_I· radicals generated by photodissociation have drastically different molecular structures [9,10,11,12]. Specifically, the C_2_H_4_I· radical has a bridged structure, providing direct structural evidence supporting the Skell hypothesis, whereas the C_2_F_4_I· radical has *classical anti* or *gauche* structures. These results demonstrate well the drastic effect of fluorination on reaction intermediates. Moreover, fluorination strongly affects the reaction pathways leading to the formation of the final products. The C_2_H_4_I· radical does not undergo direct secondary C−I bond dissociation; instead, it combines with the dissociated I· radical to form the C_2_H_4_I−I isomer, which then dissociates into C_2_H_4_ and I_2_. In contrast to C_2_H_4_I·, C_2_F_4_I· directly undergoes the secondary C−I bond dissociation to form C_2_F_4_ and I without the formation of a C_2_F_4_I−I isomer. The liberated I atoms then nongeminately recombine to form I_2_.

Besides the effect of fluorination on the structures of reaction intermediates and the reaction pathways, we aim here to investigate how fluorination affects solvent dependence by comparing the effect of solvents on the reactions of C_2_H_4_I_2_ and C_2_F_4_I_2_. Previous TRXL studies showed that the photodissociation of C_2_H_4_I_2_ is strongly affected by the solvent. In solution-phase reactions, the solvent influences the energetics and the dynamics of solute molecules through solute–solvent interactions [2,3,4,5,6,7,8,72,73]. Therefore, to understand the mechanism of a chemical reaction in solution, it is essential to consider the effect of solute–solvent interactions on the reaction mechanism [12,13,14,26,27,28,29,30]. According to a previous TRXL study of C_2_H_4_I_2_ in cyclohexane, the C_2_H_4_I· radical has a *bridged* structure as for the same reaction in methanol, but its dynamics and mechanism vary substantially depending on the type of solvent [10,11,12]. Specifically, the reaction mechanism associated with the secondary C−I bond dissociation of the C_2_H_4_I· radical, which is formed by the primary C−I bond dissociation of C_2_H_4_I_2_, highly depends on the type of solvent. Unlike in methanol, where the C_2_H_4_I· radical does not undergo direct secondary C−I bond dissociation and instead combines with the dissociated I· radical to form the C_2_H_4_I−I isomer, in cyclohexane, secondary C−I bond dissociation directly occurs to form C_2_H_4_ and I, in addition to the formation of C_2_H_4_I−I isomer, also occurring in a branched manner. In addition, C_2_H_4_I−I isomer is formed more slowly in cyclohexane than in methanol while the dissociation of C_2_H_4_I−I isomer into C_2_H_4_ and I_2_ is also more accelerated in cyclohexane than in methanol. These results suggest that the solute–solvent interaction can have a profound effect on the dynamics and mechanism of a chemical reaction. In contrast, the solvent dependence of the photodissociation of C_2_F_4_I_2_ has not yet been investigated.

To better understand the effect of fluorination and solvent dependence, it is worthwhile to compare the reaction dynamics of C_2_H_4_I_2_ and C_2_F_4_I_2_ in two different solvents. The structural dynamics of the photodissociation of C_2_F_4_I_2_ were examined by TRXL only in methanol [9,11]. Inspired by the solvent dependence observed for the photoreaction of C_2_H_4_I_2_, in this work, we used TRXL to investigate the structural dynamics of C_2_F_4_I_2_ in cyclohexane. In comparison with the TRXL results for C_2_F_4_I_2_ in methanol, the reaction mechanism of C_2_F_4_I_2_ photodissociation remains intact regardless of solvent, and the reaction parameters such as the *anti*-to-*gauche* ratio and time constants for the relevant reaction pathway are only marginally different than those for the photoreaction in methanol. Meanwhile, a comparison of bimolecular rates for nongeminate recombination to form I_2_ from the liberated iodine radicals in two solvents (cyclohexane and methanol) revealed considerable solvent dependence. We also show that the molecular structures of C_2_F_4_I_2_ and C_2_F_4_I· radicals determined from the structural analysis of TRXL data can serve as a benchmark for DFT calculations, especially for investigating the relevance of exchange-correlation functionals and basis sets used for structure optimization of haloalkanes.

## 2. Results and Discussion

### 2.1. Time-Resolved Difference Scattering Curves of C_2_F_4_I_2_ Photodissociation

**Figure 1:**
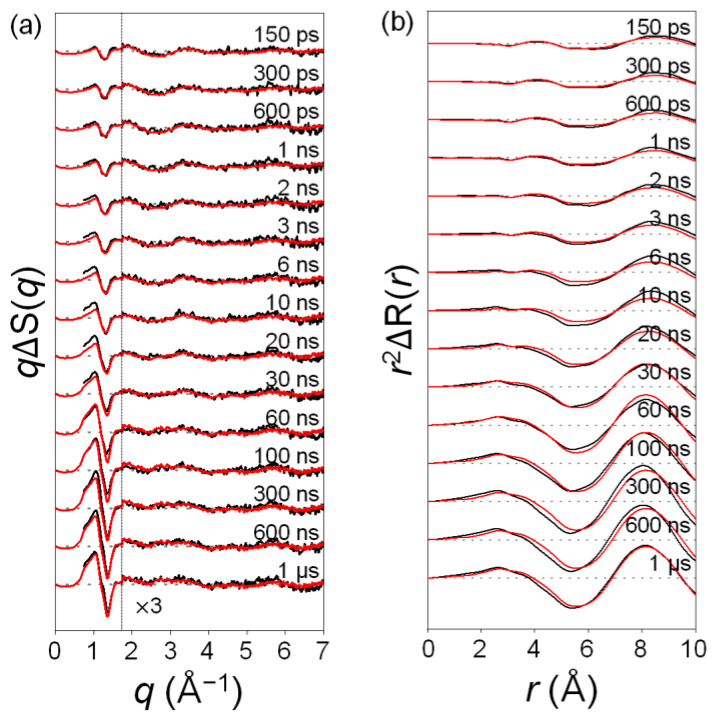
(**a**) Time-resolved difference scattering curves *q*ΔS(*q*, *t*) for C_2_F_4_I_2_ in cyclohexane as a function of time delay after photoexcitation at 267 nm. Experimental curves (black), *q*ΔS(*q*, *t*) = *q*S(*q*, *t*) − *q*S(*q*, −200 ps) are compared with calculated curves (red) obtained from global-fit analysis. Data at high *q* values (1.8 ~ 7.5 Å^–1^) were scaled up by a factor of three for better visualization. (**b**) Difference radial distribution functions (ΔRDFs), *r*^2^ΔR(*r*, *t*), obtained by the sine Fourier transformation of *q*ΔS(*q*) curves shown in (**a**).

The difference scattering curves at various time delays are shown in Figure 1a. Difference scattering curves ΔS(*q*, *t*) are multiplied by the magnitude of momentum transfer vector *q* to yield *q*ΔS(*q*, *t*). By doing so, the small difference scattering signals at high *q* values are emphasized. The *q*ΔS(*q*, *t*) curves exhibit distinct oscillatory features in *q*-space, which are the signature of structural changes of reacting molecules, and these features change with time, which indicates that reactions occur on the time scales covered by the TRXL experiment. The best-fit theoretical *q*ΔS(*q*, *t*) curves obtained from global-fitting analysis (GFA) described in Section 3.3 are shown with the experimental *q*ΔS(*q*, *t*) curves in Figure 1a. Fitting parameters from GFA are shown in Table 1. To visualize real-space information, the difference radial distribution functions (ΔRDFs), *r*^2^ΔR(*r*, *t*), where *r* is the interatomic distance, were obtained by the sine Fourier transformation of *q*ΔS(*q*, *t*), as shown in Figure 1b.

The ΔRDF signal provides the change in the distribution of interatomic distance *r* of the solute species. *r*^2^ΔR(*r*, *t*) curves exhibit distinct positive or negative peaks in terms of *r*. The positive peak indicates the formation of an atom–atom pair, whereas the negative peak shows the disappearance of an atom–atom pair, generally related to bond cleavage [12,15,16].

**Figure 2:**
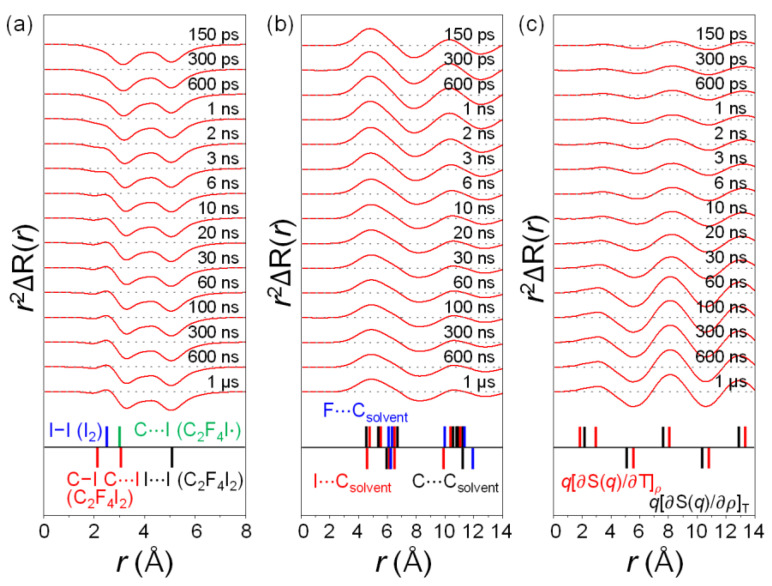
ΔRDFs represented in *r*-space are decomposed into three components: (**a**) solute-only term, (**b**) solute–solvent cross term, (**c**) solvent-only term. Lines in (**a**) correspond to the bond lengths of solute species calculated by DFT calculation. Black, red, green, and blue lines correspond to I−I of C_2_F_4_I_2_, C−I of C_2_F_4_I_2_, C−I of C_2_F_4_I· radical, and I−I of I_2_, respectively.

Because the TRXL signal is a superposition of solute, cage, and solvent terms, it is not straightforward to assign the features in *r*^2^ΔR(*r*, *t*) to specific atom–atom pairs of the chemical species. To facilitate the assignment of the features in the difference scattering curves, we decomposed it into three contributions—the solute-only term, the cage term, and the solvent-only term—as shown in Figure 2. The assigned major features of the difference scattering are indicated using the lines drawn at the bottom of each plot in Figure 2. The lines for the solute-only term were obtained from the molecular structures of the reactants, intermediates, and products, and those for the solute–solvent cross term and the solvent-only term were obtained from g(*r*)’s, calculated from the MD simulation implemented for all chemical species involved in the reaction. g(*r*) represents the distribution of the distance in an atom–atom pair. Because each line in Figure 2 has different degrees of contribution and broadening to the total ΔRDF, the positions of the peaks in the ΔRDFs might not perfectly match individual lines. The solute-only term shown in Figure 2a clearly demonstrates the structural evolution of the reacting solute molecules. For example, at 150 ps, two negative peaks at 3.1 and 5.2 Å mainly reflect the distances of the C⋯I and I⋯I atomic pairs, respectively, of depleted C_2_F_4_I_2_ parent molecules. As the reaction progressed, a positive peak at 2.7 Å grew. This peak corresponds to the I−I atomic pair of I_2_ and indicates the formation of I_2_. Besides these peaks, a negative contribution from the C−I distance (2.1 Å) of the C_2_F_4_I_2_ molecule and a positive contribution from the C⋯I distance (3.0 Å) of C_2_F_4_I· were present, but were hidden by other features of larger amplitudes and broadenings.

Information on the solvent environment around the solutes can also be obtained by the solute–solvent cross-term in Figure 2b. In particular, the C and H atoms of the cyclohexane solvent engaged in long-range interactions with the C, H, F and I in the solute molecules. ΔRDF reflects the time-resolved concentration of these atomic pairs at each distance, and the atomic pairs containing heavy atoms such as I largely contribute due to their large atomic form factors. For example, two positive peaks at 5.0 and 10.4 Å, and a negative peak at 8.0 Å are distinct at 150 ps. The positive peaks imply the emergence of a new atomic pair, whose peak positions match with the distances between the atoms of C_2_F_4_I· and the C atom in cyclohexane. On the other hand, the negative peak at 8.0 Å corresponds to the distance between the I atom in the consumed C_2_F_4_I_2_ and two H atoms in cyclohexane. As the reaction progressed, the positive peaks shifted to 4.8 and 10.6 Å, while a negative one elongated to 8.2 Å at 100 ns with modified peak amplitudes. They represent the dynamic rearrangement of the solvent cage structure in response to the formation and dissociation of the later intermediates.

From the solvent-only term in Figure 2c, one can obtain information on heat dissipation and subsequent solvent rearrangement induced by photoexcitation and photoreaction. The difference scattering of the solvent consisted of the *q*(∂S(*q*)/∂T)*_ρ_* and *q*(∂S(*q*)/∂*ρ*)_T_ terms. The *q*(∂S(*q*)/∂T)*_ρ_* term was responsible for the increase in temperature (and pressure) of the solvent at a constant volume, which occurred at the early stage of the reaction (<6 ns). The *q*(∂S(*q*)/∂*ρ*)_T_ term accounted for the thermal expansion that occurred after 6 ns. The expansion led to the equilibration with an ambient pressure and the decrease of the solvent density. As a result, the C⋯C distances in adjacent cyclohexane molecules changed, resulting in highly oscillatory features in the difference scattering curves after 6 ns.

### 2.2. Determination of the Structure of the Radical Intermediate

**Figure 3:**
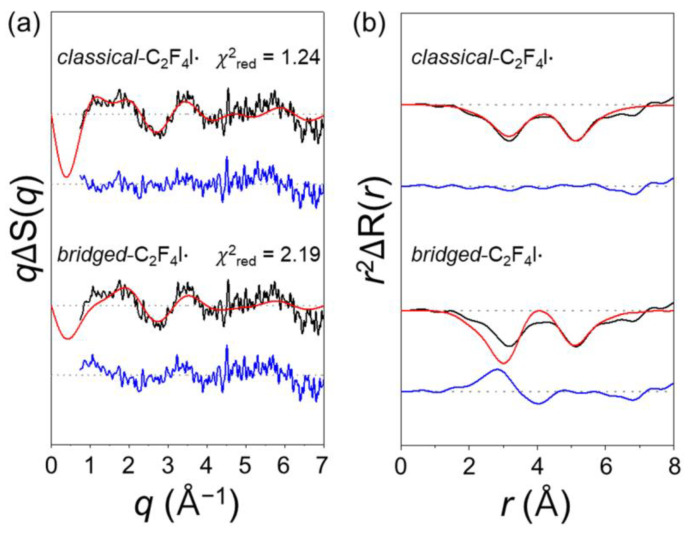
Comparison of models using only either *classical* (top) or *bridged* (bottom) structure of C_2_F_4_I· radical. The contribution only associated with the formation of C_2_F_4_I· radical (C_2_F_4_I_2_ → C_2_F_4_I· + I·) was extracted from experimental data at 100 ps (and its theoretical fit) by subtracting the cage and solvent terms, and the contribution of other solute species. The extracted experimental (black) and theoretical (red) scattering curves for the formation of C_2_F_4_I· only are compared in the (**a**) *q*-space and (**b**) *r*-space. Blue curve represents the obtained residual by subtracting the theoretical curve from the experimental curve. The reduced chi-squared value for each fit is shown above the experimental and theoretical curves. The *classical* structure gave a much better fit than the *bridged* structure does.

The structure of the haloethyl radical has been under debate between the *classical* and *bridged* forms [46,47,48,49]. TRXL studies on the photodissociation of C_2_H_4_I_2_ and C_2_F_4_I_2_ directly revealed that the C_2_H_4_I· and C_2_F_4_I· radicals have the *bridged* structure and the *classical* open structure, respectively [9,12]. These studies underscore the influence of fluorination on the molecular structure and reaction kinetics. To examine if the radical structure is influenced by the polarity of the solvent, we performed GFA with the structure of C_2_F_4_I· as (ⅰ) a *classical* structure, that is, a mixture of *anti*-C_2_F_4_I· and *gauche*-C_2_F_4_I· or (ⅱ) a *bridged* structure. To better structurally distinguish between the two radical structures, we carefully extracted only the contribution related to the C_2_F_4_I_2_ → C_2_F_4_I· + I· pathway. To do so, we subtracted the contributions of solvent, cage, and other solute species from the data at 150 ps. The extracted contributions of only C_2_F_4_I· for the *bridged* and *classical* models are shown in Figure 3. The negative peak at 5.2 Å corresponds to the I⋯I distance of the depleted parent molecule and is common for both models. However, the shapes of the peak in the *r*-range of 2.0–4.5 Å are quite different from each other in the two models. This region corresponds to the distances of the I atom relative to two carbon and four fluorine atoms in the radical. The *classical* structure has two C−I distances and two (*anti*) or three (*gauche*) F-to-I distances, whereas the *bridged* structure has only one C−I distance and one F−I distance due to its symmetric geometry. Therefore, the difference in peak shape in this region serves as a fingerprint of the *classical* structure. The *classical* model fits the experimental data at 150 ps better than the *bridged* model does. To quantify the fitting quality between the two models, χ^2^*_class_*/χ^2^*_bri_*, the ratio of the reduced chi-squared values between the *classical* and *bridged* models was calculated at each time delay from the best-fit result for each model (Figure S2). The ratio is significantly lower than 1 at 150 ps, where the concentration of the C_2_F_4_I· radical is high. As the concentration of C_2_F_4_I· radical decreases at later time delays, the ratio expectedly approaches 1. Therefore, the *classical* radical gives a better fit to the experimental data than the *bridged* radical does. In fact, when we include a mixture of *bridged* and *classical* structures in the fitting with their concentration ratio as a variable, the concentration of the *bridged* radical converges to zero.

When the structures of C_2_F_4_I· radical and C_2_F_4_I_2_ parent molecule calculated from the DFT calculation using the ωB97X functional as the DFT exchange-correlation functional were used without any alteration (as listed in Table S2), the fit was already quite good. This result means that the DFT structures calculated using the ωB97X functional were accurate. The advantage of the ωB97X functional for predicting accurate structures in halomethanes and haloethanes was also reported for the TRXL studies of C_2_H_4_I_2_ in cyclohexane [12] and CHI_3_ in cyclohexane [15], which clearly demonstrates the excellent agreement between the DFT-optimized structures (except for that of *iso*-CHI_2_−I) calculated using ωB97X functional and the experimentally determined structures. The structural parameters of CF_3_Br, optimized by using the ωB97X functional, are in excellent agreement with the experimentally determined parameters from the analysis of microwave spectra [45]. Nevertheless, we refined the structures of *anti*-C_2_F_4_I·, *gauche*-C_2_F_4_I·, *anti*-C_2_F_4_I_2_, and *gauche*-C_2_F_4_I_2_ by varying the C−I distances and CCI angles to obtain better agreement between the experimental and calculated curves (Table 2). In the previous TRXL study of C_2_F_4_I_2_ in methanol, the structures from DFT calculations were used for data analysis without any refinement.

The refined structures of *anti*-C_2_F_4_I·, *gauche*-C_2_F_4_I·, *anti*-C_2_F_4_I_2_, and *gauche*-C_2_F_4_I_2_ can serve as an experimental standard for benchmarking various combinations of correlation-exchange functionals and basis sets. We performed DFT calculations using five functionals (ωB97X, M06-2X, B3LYP-D3, PBE0, and TPSSh) and three basis sets (def2-TZVPP, cc-pVTZ(-PP), and aug-cc-pVTZ(-PP)). Then, we compared the root-mean-square deviation values for the C−I distances and CCI angles as shown in Table 3. This comparison immediately confirmed that the ωB97X functional had the best agreement with the experimentally determined structure, regardless of the basis set.

We also tested the possibility of the formation of the C_2_F_4_I−I isomer. In the photoinduced iodine elimination reaction of C_2_H_4_I_2_, the formation of the C_2_H_4_I−I isomer by the addition of I· to C_2_H_4_I· served as a critical route to generate the final products, C_2_H_4_ and I_2_. In contrast, the C_2_F_4_I−I isomer was not observed in the photoreaction of C_2_F_4_I_2_ in methanol. To examine if the isomer was formed in a different solvent, cyclohexane, we performed GFA with the reaction pathway involving the C_2_F_4_I−I isomer. The inclusion of the isomer in the reaction mechanism significantly increased the χ^2^_red_ value by a factor of 1.3, confirming that such an isomer was not observed in the TRXL data. In terms of relative energies, the results of the DFT calculation show that the energy of the C_2_F_4_I−I isomer relative to C_2_F_4_I· + I· is −132.4 kJ/mol and −132.5 kJ/mol in cyclohexane and methanol, respectively. Therefore, the consideration of the energetics alone does not exclude the formation of the C_2_F_4_I−I isomer. For the sake of comparison, the energy of the C_2_H_4_I−I isomer relative to C_2_H_4_I· + I· was −137.5 and −134.6 kJ/mol in cyclohexane and methanol, respectively.

### 2.3. Kinetics and Mechanism of C_2_F_4_I_2_ Photodissociation

**Figure 4:**
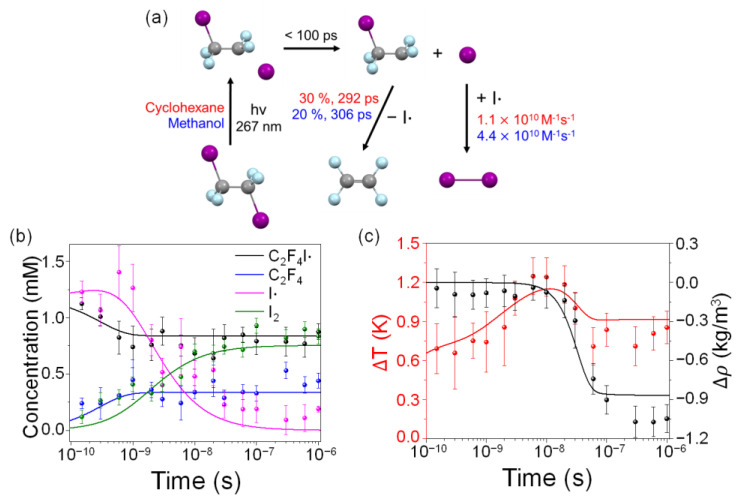
(**a**) Reaction mechanism of C_2_F_4_I_2_ photodissociation in cyclohexane determined in this study. Kinetic parameters for the reactions in cyclohexane and methanol are marked in red and blue, respectively. (**b**) Time-dependent concentration changes of chemical species involved the photodissociation reaction of C_2_F_4_I_2_ in cyclohexane. (**c**) Time-dependent changes of solvent temperature (red) and density (black) induced by photodissociation of C_2_F_4_I_2_. Solid lines were obtained from optimized global fits based on kinetic models, and symbols were obtained from individual fits of experimental difference scattering curves at various time delays.

The reaction mechanism of C_2_F_4_I_2_ photodissociation is shown in Figure 4a, and the time-dependent concentration changes of each chemical species obtained from the GFA are shown in Figure 4b. The rate constants and branching ratios for all reaction pathways are summarized in Table 1. For comparison, the kinetic information for C_2_F_4_I_2_ photodissociation in methanol adapted from the previous TRXL study is also indicated in Figure 4a, and the kinetic parameters obtained from the global fitting of the data in methanol from the earlier work are listed together in Table 1. Upon photoexcitation, the C_2_F_4_I_2_ molecule loses one iodine atom, forming C_2_F_4_I· earlier than 150 ps, which is the earliest time delay. Such ultrafast photodissociation of a halogen atom is a common feature observed in haloalkanes in various solvents [12]. The 30 ± 1.1% of the C_2_F_4_I· radical undergoes secondary dissociation, C_2_F_4_I· → C_2_F_4_ + I·, with a rate constant of 3.44 ± 2.9 × 10^9^ s^−1^ (corresponding to a time constant of 292 ± 34 ps). The two I· radicals undergo nongeminate recombination with each other to form molecular iodine in tens of nanoseconds, with the bimolecular rate constant of 1.1 ± 0.8 × 10^10^ M^−1^s^−1^.

Besides the concentration dynamics of the solute species, we could also obtain information on the dynamics of heating and the expansion of the bulk solvent. When the reactant molecules were photoexcited by laser pulses, a fraction of molecules (7% in this case) rapidly recovered back to the ground state by geminate recombination and vibrational cooling in the ground state, thus dissipating heat to the environment. As a result, the temperature and density of the solvent in the laser focal volume were changed, as shown in Figure 4c. At early time delays up to 10 ns, heat was dissipated at a constant volume, leading to an increase in temperature by a total of 1.15 K at 10 ns, as described by the solvent differential of *q*(∂S(*q*)/∂T)*_ρ_*. After 10 ns, thermal expansion occurred, leading to a decrease in solvent density by a total of −0.86 kg/m^3^ at 1 μs, with a 50 ns time constant. With the expansion, the solvent temperature also decreased, giving a total temperature change of 0.9 K at 100 ns. These changes in density and temperature accompanying the photodissociation of C_2_F_4_I_2_ in cyclohexane fell into the typical range observed in other TRXL experiments, as shown in Table S1.

### 2.4. Solvent Dependence of Reaction Dynamics

We examined the solvent dependence of the reaction dynamics by comparing how the same solute, C_2_F_4_I_2_, evolves into two different solvents, cyclohexane and methanol. TRXL studies on the photodissociation of C_2_H_4_I_2_ in cyclohexane and methanol provide a useful example to discuss the solvent dependence of solution-phase reactions of haloalkanes. C_2_H_4_I· in cyclohexane only follows a pathway to form the C_2_H_4_I−I isomer, which then decays into C_2_H_4_ and I_2_, thus lacking a pathway of direct dissociation of C_2_H_4_I· to form C_2_H_4_ and I· observed in methanol. Moreover, the lifetime of the C_2_H_4_I−I isomer is shorter in cyclohexane than that in methanol. These solvent dependences were explained on the basis of solvent polarity that could significantly affect the rates and pathways of a chemical reaction. Accordingly, we compared the photodissociation mechanism of C_2_F_4_I_2_ in cyclohexane with the published results on the same molecule in methanol [9] to depict the solvent dependence of the photodissociation of the tetrafluorinated derivative of C_2_H_4_I_2_. In detail, we focused on how the following measurables vary depending on the solvent environments: (1) the *anti*-to-*gauche* ratio of C_2_F_4_I·; (2) the structural conformation of C_2_F_4_I· (*classical* vs *bridged*); (3) the secondary dissociation kinetics from C_2_F_4_I· to C_2_F_4_; (4) the recombination rate of two I· into a molecular iodine.

#### 2.4.1. Anti-to-Gauche Ratio of C_2_F_4_I·

First, the GFA of our TRXL data yielded 84(±3.7):16 for the *anti*-to-*gauche* ratio of C_2_F_4_I· in cyclohexane, which was comparable to 86(±4.2):14 reported by the analogous study in methanol. From the DFT calculations, the free-energy gap between the two conformers of C_2_F_4_I· was determined to be 9.9 kJ/mol in cyclohexane and 9.5 kJ/mol in methanol. The corresponding *anti*-to-*gauche* conformer ratios are 98:2 in both cyclohexane and methanol, which are much larger than the ratios of 84:16 and 86:14 determined from the TRXL measurement. This discrepancy indicates that the DFT calculations overestimated the energy difference between *anti* and *gauche* conformers. The observed ratios of 84:16 in cyclohexane and 86:14 in methanol gave estimated free-energy differences of 4.1 and 4.5 kJ/mol, respectively. These values were smaller than the DFT values by ~50%, which indicated that the DFT-calculated energy differences were overestimated by about a factor of 2. The DFT-calculated energy gap between the two conformers of C_2_F_4_I· was larger in cyclohexane by only 0.4 kJ/mol than that in methanol. If we applied the estimated scaling factor, the free-energy difference was estimated to be 0.2 kJ/mol, which was negligibly small. Therefore, the nearly identical *anti*-to-*gauche* conformer ratios of C_2_F_4_I· in cyclohexane and methanol were not surprising.

#### 2.4.2. Structural Conformation of C_2_F_4_I·

Generated haloethyl radicals C_2_F_4_I· and C_2_H_4_I· follow different fates. First, in both cyclohexane and methanol, C_2_H_4_I· predominantly exists as a *bridged* conformer. In contrast, C_2_F_4_I· favors *classical* (*anti* and *gauche*) conformations in both cyclohexane and methanol, as discussed in the previous section. In the photoreaction of C_2_H_4_I_2_, the *bridged*-C_2_H_4_I· radical binds with the initially dissociated I· to form C_2_H_4_I−I isomer in hundreds of picoseconds, and subsequently dissociates into C_2_H_4_ and I_2_ in both methanol and cyclohexane. The C_2_H_4_I−I isomer exists as an intermediate in both cyclohexane and methanol and is energetically stable. According to ωB97X/def2-TZVPP, the Gibbs free energy of the C_2_H_4_I−I isomer is 28.3 kJ/mol in cyclohexane and 32.0 kJ/mol in methanol with respect to *anti*-C_2_H_4_I_2_, and the Gibbs free energy of C_2_H_4_ + I_2_ is 11.0 kJ/mol in cyclohexane and C_2_H_4_ + I_2_ is 13.5 kJ/mol in methanol (Figure S6). The enthalpies of C_2_H_4_ + I_2_ were actually higher than those of the C_2_H_4_I−I isomer in both cyclohexane and methanol, indicating that the dissociation of C_2_H_4_I−I into C_2_H_4_ and I_2_ is entropy-driven. In cyclohexane, the direct secondary dissociation from C_2_H_4_I· to C_2_H_4_ + I· also occurred as a bypath that accounted for 52% of the total yield of C_2_H_4_. In contrast, the C_2_F_4_I−I isomer was not observed.

#### 2.4.3. Secondary Dissociation Kinetics from C_2_F_4_I· to C_2_F_4_

The secondary dissociation from C_2_F_4_I· to C_2_F_4_ occurs only via the direct loss of an additional I·, unlike in the case of C_2_H_4_I·. The C_2_F_4_I−I isomer is not formed at all. According to a previous TRXL study, C_2_F_4_I· decays to form its final product, C_2_F_4_, with a time constant of 306 ps in methanol. This time constant is nearly the same as that of 292 ps in cyclohexane within the experimental uncertainty. In addition, the dissociating portion of the radical is exposed to a subtle change from 20 ± 1.3% in methanol to 30 ± 1.1% in cyclohexane. Such negligible solvent dependence is in stark contrast to the strong solvent dependence observed for C_2_H_4_I. To explain such different degrees of solvent dependence of C_2_F_4_I_2_ and C_2_H_4_I_2_, we compared the dipole moments of the C_2_F_4_I· (*anti*-C_2_F_4_I·) and C_2_H_4_I· (*bridged*-C_2_H_4_I·) radicals. According to DFT calculations, the dipole moment of C_2_F_4_I· (μ = 0.42 D) was significantly smaller than that of C_2_H_4_I· (μ = 1.68D). Generally, a polar solute species should be relatively more stabilized in a polar solvent (methanol) than in a nonpolar solvent (cyclohexane), with the degree of stabilization proportional to the dipole moment of the solute species. Thus, the observed that the difference in the degrees of solvent dependence of C_2_F_4_I·and C_2_H_4_I·can be attributed to the difference in the dipole moments of those radical intermediates.

#### 2.4.4. Recombination Rate of Two I into I_2_

After secondary dissociation, the two I· radicals nongeminately recombined to form I_2_ with a bimolecular reaction rate constant of 1.1 ± 0.8 × 10^10^ M^−1^s^−1^ in cyclohexane. This rate constant was smaller than the reported rate constant of 4.4 ± 1.3 × 10^10^ M^−1^s^−1^ for the analogous reaction in methanol, indicating that the nongeminate I_2_ formation during the photodissociation of C_2_F_4_I_2_ is slower in cyclohexane than that in methanol. The bimolecular rate constants are expected to depend on the type of solvent rather than the parent molecule. Indeed, according to the TRXL measurements on various solutes in cyclohexane, the bimolecular rate constants for the formation of I_2_ were in the range of 0.65–1.58 × 10^10^ M^−^^1^s^−^^1^, and those in methanol were in the range of 3.1−4.4 × 10^10^ M^−^^1^s^−^^1^, as shown in Table S1. This trend can be explained by considering the solvent viscosity. The bimolecular recombination requires the two I· radicals to move into close vicinity of each other. Therefore, the bimolecular recombination rate is limited by diffusion in a solution. Considering that both reactant (I· radicals) and product (I_2_) are nonpolar, the diffusion is slower in a more viscous solvent. In fact, the recombination of I_2_ is slower in a solvent of higher viscosity [76]. The diffusion rate (*k*_D_) can be estimated via the following equation derived from the Fick’s law of diffusion and the Stokes–Einstein equation:

*k*_D_ = 8*RT*/3*η* (1)

where *R* is the gas constant, *T* is the temperature, and *η* is the viscosity of a solvent. This equation shows that *k*_D_ is inversely proportional to the solvent viscosity. The viscosities of cyclohexane and methanol are 0.89 and 0.54 cP, and the corresponding *k*_D_ values are 7.3 × 10^9^ and 1.18 × 10^10^ M^−1^s^−1^, respectively [77]. The relative magnitudes of *k*_D_ values in cyclohexane and methanol agree with the experimental observation that the bimolecular rate in cyclohexane is smaller than that in methanol, although absolute values show discrepancy against the measured bimolecular rates. The difference in absolute scale stems from rough approximations. For example, the shape of diffusing species, the interatomic forces, and the intrinsic reaction rate during the derivation, which can affect the exact estimation result, are ignored here. The same trend related with the viscosity was also observed in the nongeminate recombination of Br· radicals to form Br_2_ in the photoreaction of HgBr_2_ in acetonitrile and methanol, which was studied with TRXL. The bimolecular recombination rate constants to form Br_2_ are 2 ± 1×10^10^ M^−1^s^−1^ in acetonitrile [56] and 8.5 ± 0.1 ×10^9^ M^−1^s^−1^ in methanol [74]. The relative magnitudes of the bimolecular rate constants are consistent with those of the viscosities; the viscosity of acetonitrile (0.34 cP) is smaller than that of methanol (0.54 cP).

### 2.5. Various Types of Solvent Dependences

**Figure 5:**
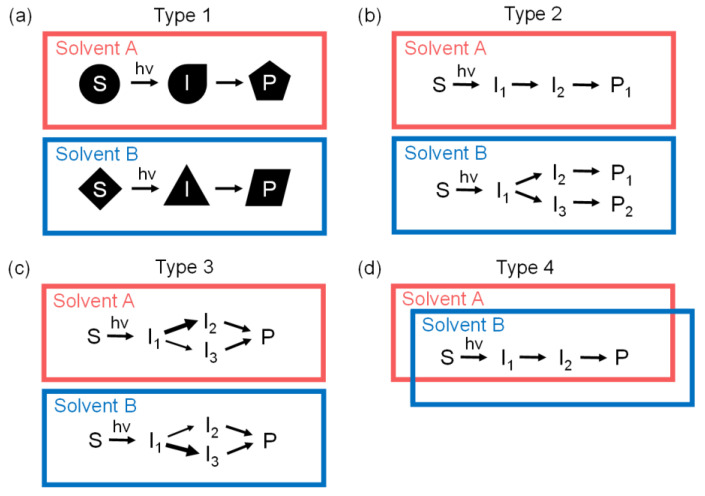
Four different types of solvent dependence of photoreactions. (**a**) In Type 1, the molecular structures of reactants, reaction intermediates, and/or products vary significantly depending on solvent. (**b**) In Type 2, part of the reaction pathway is altered depending on the solvent. (**c**) In Type 3, only the rates or branching ratios of some reaction steps are affected by the change in solvent, while the overall framework of the chemical reaction is maintained. (**d**) In Type 4, no significant differences are observed in different solvents.

Solvent dependence has been investigated for various chemical reactions, and different types of solvent dependences have been reported. The observed solvent dependence can be classified into four types as shown in Figure 5. In Type 1, the molecular structures of reactants, reaction intermediates, or products significantly vary depending on the solvent, as can be seen in the reactions of I_3_^−^ [51,75] and I_2_ [72]. For example, I_3_^−^ adopts solvent-dependent molecular structures, a symmetric structure in acetonitrile and an asymmetric structure in water. In Type 2, part of the reaction pathway is altered depending on the solvent, as can be seen in the photoreactions of CH_2_I_2_ [6,13], CH_2_IBr [14], CHBr_3_ [30], CHI_3_ [5,15], and HgI_2_ [55,56]. In the case of the photodissociation of CHI_3_, an isomerization pathway leading to the formation of iso-CHI_2_−I is active in cyclohexane, whereas the pathway is inactive in methanol. C_2_H_4_I_2_ also belongs to this type, as the direct dissociation pathway, C_2_H_4_I· → C_2_H_4_ + I, is blocked in methanol and activated in cyclohexane. In Type 3, only the rate or branching ratio of some reaction steps is affected by the change in solvent, while the overall framework of the chemical reaction is maintained, as can be seen in the photoreactions of HgBr_2_ [56,74], CF_2_I_2_ [39,44], and Fe_3_(CO)_12_ [78]. For the photodissociation of CF_2_I_2_, the rate constants for some reaction pathways are different in two different solvents, carbon tetrachloride and cyclohexane, while the entire photodissociation pathways are common for the reactions in the two solvents. In Type 4, no significant differences are observed in different solvents, as can be seen in the photoreactions of anthracene [79,80] and 1-phenyl pyrene [81]. For example, the excited-state dynamics of 1-phenyl pyrene is almost the same in different solvents. The photodissociation of C_2_F_4_I_2_ investigated in this work belongs to Type 4.

## 3. Materials and Methods

### 3.1. Time-Resolved X-ray Liquidography Experiment

The TRXL experiment was conducted with the pump-probe scheme at the NW14A beamline of the High-Energy Research Organization (KEK). The detailed setup of the TRXL experiment is described in the literature [51,82,83]. Briefly, a 60 mM solution of C_2_F_4_I_2_ (Apollo Scientific, 97%) in cyclohexane was circulated through a sapphire jet nozzle to form a 300 μm thick liquid sheet. Femtosecond optical laser pulses with a center wavelength of 267 nm were used to excite molecules in solution. The laser beam was focused to a spot size of 520 × 310 μm^2^ at the stable part of the liquid jet where the laser beam overlapped with the X-ray beam at the crossing angle of 10°. At the sample position, the laser fluence was 1.19 mJ/mm^2^. The pink X-ray beam (ΔE/E = 4.875%) with 3 × 10^8^ photons per pulse and the center wavelength of 0.7114 Å were employed to probe the dynamics of the sample. The X-ray spot size at the sample position was 200 × 200 μm^2^.

The scattering patterns were collected by an area detector (MarCCD 165) placed at 31.34 mm apart from the sample position at the following pump-probe time delays: −200 ps, 150 ps, 300 ps, 600 ps, 1 ns, 2 ns, 3 ns, 6 ns, 10 ns, 20 ns, 30 ns, 60 ns, 100 ns, 300 ns, 600 ns, and 1 μs. The scattering patterns measured at −200 ps were used as a reference to account for the sample before the photoreaction and to generate difference scattering curves *q*ΔS(*q*, t). The scattering signal due to the heating of the cyclohexane solvent was obtained by performing a separate experiment at two time delays (150 ps and 1 μs).

### 3.2. Data Processing

One-dimensional (1D) scattering curves S(*q*, *t*) were obtained by azimuthal integration of the two-dimensional (2D) scattering images as a function of the magnitude of momentum transfer, *q* = (4π/*λ*)sin(*θ*), where *λ* is the wavelength of X-rays, 2*θ* is the scattering angle, and *t* is the time delay between laser and X-ray pulses. The intensities of scattering curves were normalized by the sum of intensity divided by the number of *q* points within 4 to 7 Å^–1^, and scaled to the absolute scale of the total (elastic and inelastic) scattering of one cyclohexane molecule. After scaling the intensities, difference scattering curves *q*ΔS(*q*, *t*) were obtained by subtracting the scattering curve at a negative time delay (*t* = −200 ps) from the scattering curves at positive time delays and multiplying *q* to amplify the intensities at large scattering angles. The difference radial distribution functions (RDFs), *r*^2^Δ*R*(*r*, *t*), were obtained by the sine Fourier transform of the *q*Δ*S*(*q*, *t*) curves as reported in previous publications [11,51,52].

### 3.3. Data Analysis

The theoretical difference of X-ray scattering curves ΔS(*q*, *t*)_theory_ of the solution sample included three components, the (ⅰ) solute-only term, (ⅱ) solute–solvent cross term, and (ⅲ) solvent-only term, which were computed as reported in previous publications [15,51]. The solute-only term was calculated using the Debye equation. The solvent-only term ΔS(*q*, *t*)_solvent_ consisted of two differentials, (∂S/∂T)*_ρ_* and (∂S/∂*ρ*)_T_, and was expressed as follows:

ΔS(*q*, *t*)_solvent_ = ΔT(*t*) × (∂S/∂T)*_ρ_* + Δ*ρ*(*t*) × (∂S/∂*ρ*)_T_ (2)

where (∂S/∂T)*_ρ_* is the change in solvent scattering intensity in response to a temperature change at a constant density, and (∂S/∂*ρ*)_T_ is the change in solvent scattering intensity in response to a density change at a constant temperature. ΔT(*t*) and Δ*ρ*(*t*) are the time-dependent changes in the temperature and density of the solvent, respectively. Differentials (∂S/∂T)*_ρ_* and (∂S/∂*ρ*)_T_ for the solvent were determined from a separate measurement on a dye, 4-bromo-4′-(*N*,*N*-diethylamino)-azobenzene, dissolved in cyclohexane at 7.18 mM concentration to have the same optical density as that of the sample solution. In addition to these two differentials for the solvent, we also considered artifacts arising from the response of the cyclohexane solvent under a strong laser fluence. Solute–solvent cross term ΔS(*q*, *t*)_cage_ was calculated from the molecular-dynamics (MD) simulation for all the chemical species in the reaction. Therefore, theoretically constructed scattering curves S(*q*)_theory_ were convoluted with the measured X-ray spectrum to take this polychromaticity into account. Difference scattering curves *q*ΔS(*q*, *t*) were examined by global-fit analysis (GFA), a weighted least-squares method that minimizes the reduced chi squared (χ^2^_red_) between theoretical and experimental data, which is defined as follows [69,70,71]:

(3) $$\chi_{\mathrm{red}}^{2}=\frac{1}{N-p-1}\underset{j=\mathrm{time}\;\mathrm{delay}}{\sum }\underset{i}{\sum }\frac{{\left(\Delta S{\left(q_{i},t_{i}\right)}_{\mathrm{theory}}-\Delta S{\left(q_{i},t_{i}\right)}_{\exp}\right)}^{2}}{\sigma_{ij}^{2}}$$

where *N* is the total number of data points along the *q* and *t* axes, *p* is the number of fitting parameters, ΔS(*q_i_*, *t_j_*)_exp_ is the experimentally measured difference scattering intensity at the *i*th *q* and *j*th time delays, and *σ_i_*_,*j*_ is the standard deviation of the difference scattering intensity at the *i*th *q* and *j*th time delays. The χ^2^_red_ minimization was performed using the MINUIT package written at CERN, and error analysis was performed by MINOS, a built-in algorithm in the MINUIT software [84].

We retrieved the reaction rate constants of each step, the excitation fraction of C_2_F_4_I_2_, the fraction of the excited molecules relaxing back to the ground state, the *anti*-to-*gauche* conformer ratio of C_2_F_4_I, and the structural parameters and relative enthalpy of major intermediates from the analysis. The C−I bond lengths and C−C−I angles of the two conformers of C_2_F_4_I_2_ and C_2_F_4_I, in addition to the I−I bond length of I_2_, were selected as the structural degrees of freedom. The other structural parameters, such as the bond lengths of C−C and C−F, were fixed to the values from the DFT calculation to avoid possible overfit. For the enthalpy of major intermediates, only the relative enthalpy of I_2_ with respect to iodine radicals was used as a fitting parameter to fit the experimentally observed thermodynamic response of solvents. The enthalpies of other reaction intermediates were fixed to the values from the DFT calculation to avoid any potential overfit.

### 3.4. DFT Calculation

The energies and structures of C_2_F_4_I_2_, and its related species such as C_2_F_4_I· and C_2_F_4_, calculated with DFT methods were already reported for both gas and solution phases [2,25]. In a previous study, hybrid functionals (B3LYP, B3PW91, PBE0, X3LYP) and a hybrid meta functional (M05-2X) were used for the DFT calculation. In subsequent TRXL studies of photoreactions of CHI_3_ [15] and C_2_H_4_I_2_ [12], various functionals (ωB97X, B3LYP, M05-2X, M06-2X, etc.) were tested for structure optimization, and the ωB97X functional could accurately predict the C−I distance in halomethanes and haloethanes [85]. Therefore, using Q-Chem [86], we performed DFT calculations on all relevant species involved in the photodissociation of C_2_F_4_I_2_ in the gas phase, the polar solvent (methanol, ε = 32.63), and the nonpolar solvent (cyclohexane, ε = 2.023) with ωB97X as the DFT exchange-correlation functional and def2-TZVPP as the basis set. To consider the solvent environment, the conductor-like polarizable continuum model (CPCM) [87,88] method was used. For comparison, we also performed the same calculations on all relevant species involved in the photodissociation of C_2_H_4_I_2_. The calculated Gibbs free energies, including the energies of relevant species, are shown in Figure S5 (for C_2_F_4_I_2_) and Figure S6 (for C_2_H_4_I_2_). In addition, for comparison with the experimentally determined structures, we performed DFT calculations with M06-2X [89], B3LYP-D3 [90,91,92], PBE0 [93] and TPSSh [94] functionals for the relevant species involved in the photodissociation of C_2_F_4_I_2_ in cyclohexane. We used def2-TZVPP, cc-pVTZ(-PP), and aug-cc-pVTZ(-PP) basis sets, which are of triple-ζ quality [95]. The iodine was modelled with def2-TZVPP, cc-pVTZ-PP, and aug-cc-pVTZ-PP basis sets including the effective core potentials, and the other atoms were modelled with def2-TZVPP, cc-pVTZ, and aug-cc-pVTZ basis sets. The structures of all relevant species were fully optimized in the nonpolar solvent (cyclohexane, ε = 2.023), and frequency calculations were implemented for the optimized structures. The key structural parameters of the optimized structures of the chemical species obtained with various functionals are shown in Table 2. The root-mean-square deviation (RMSD) values for the structures calculated with various functionals and basis sets are listed in Table 3. The detailed structural parameters obtained from the structures optimized with various functionals and basis sets are also listed in Tables S2 and S3.

The Gibbs free energy of each optimized structure, *G*_sol_, was calculated by the following equations:

*G*_sol_ = *G*_gas_ + *G*_solv_ (4)

*G*_gas_ = *H*_gas_ − *T*·*S*_gas_ (5)

*H*_gas_ = *E*_SCF_ + ZPE (6)

where *G*_gas_ is the Gibbs free energy of the optimized structure; *G*_solv_ is the solvation free energy; *H*_gas_ is the enthalpy in the optimized structure with the *PV* term, which is about 0.59 kcal/mol at 298.15 K, being ignored; *T* is the temperature (298.15 K); and *S*_gas_ is the entropy in the optimized structure. The solvent entropies are implicitly contained in *G*_solv_ in the continuum model in Equation (4); *E*_SCF_ is the electronic self-consistent field energy computed from the SCF convergence in the optimized structure, and ZPE is the vibrational zero-point energy. The entropy in Equation (5) corresponds to the vibrational, rotational, and translational entropies of the solute(s), except that the single-atom (iodine) has only the translational entropy, which were implemented form the Sackur–Tetrode equation [96].

The diagrams including the relative Gibbs free energies, Δ*G*_sol_, and enthalpies, Δ*H*_gas_, for the reaction channels are shown in Figure S5 (for the photodissociation of C_2_F_4_I_2_) and Figure S6 (for the photodissociation of C_2_H_4_I_2_). Δ*G*_sol_ and Δ*H*_gas_ were calculated by the following equations.

Δ*G*_sol_ = Σ*G*_sol_ for products − Σ*G*_sol_ for reactants (7)

Δ*H*_gas_ = Σ*H*_gas_ for products − Σ*H*_gas_ for reactants (8)

## 4. Conclusions

**Figure 6:**
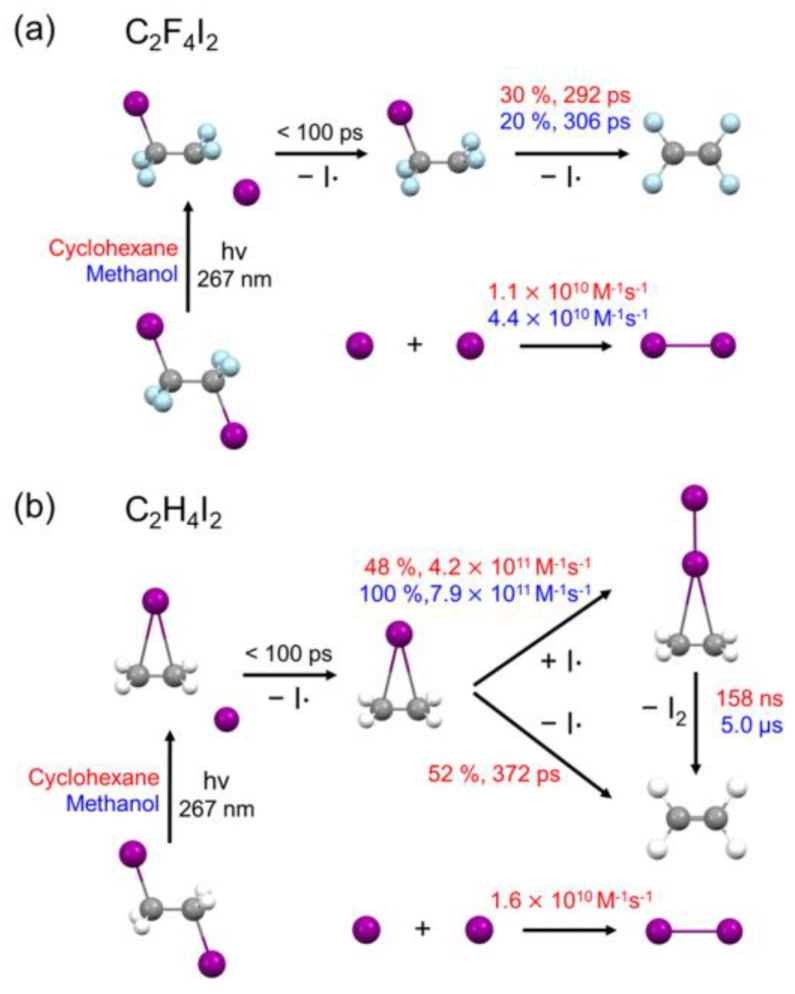
Comparison of photodissociation dynamics of (**a**) C_2_F_4_I_2_ and (**b**) C_2_H_4_I_2_ in cyclohexane and methanol. Kinetic parameters in cyclohexane and methanol are shown in red and blue, respectively. While the reaction pathways and the associated kinetics of C_2_H_4_I_2_ photodissociation substantially vary depending on the solvent, the photodissociation of C_2_F_4_I_2_ did not exhibit such strong dependence. For both C_2_F_4_I_2_ and C_2_H_4_I_2_, the structures of the reaction intermediates did not change much with the change in solvent.

In this work, we investigated the kinetics and structural dynamics of the photodissociation of C_2_F_4_I_2_ in cyclohexane, and compared them with those in methanol. We also compared the solvent dependence of the overall photodissociation dynamics of C_2_F_4_I_2_ and its defluorinated homolog, C_2_H_4_I_2_, from previous works (Figure 6). This comparative investigation revealed that fluorination greatly affects the solvent dependence and overall reaction pathways, and the molecular structure of the reaction intermediates (C_2_F_4_I· and C_2_H_4_I· radicals). The quite different structures of C_2_H_4_I· and C_2_F_4_I· radicals, and the resulting difference in the permanent dipole moments, account for the observed difference, underscoring the influence of fluorination in chemistry. Moreover, it was confirmed that the nongeminate recombination rate to form I_2_ is slower in cyclohexane than in methanol. In this work, we also emphasized the practical usefulness of the experimentally determined molecular structures by showing that they can be used to evaluate the accuracy of DFT calculations performed with various combinations of functionals and basis sets. This study demonstrates that TRXL is a powerful technique to study solvent dependence in the solution phase. Further studies for a wider range of molecules and reactions could open the door for the better understanding and control of solution-phase reactions.

## Figures and Tables

**Table 1:** Fitting parameters obtained from global fitting analysis of C_2_F_4_I_2_ in cyclohexane in comparison with those from C_2_F_4_I_2_ in methanol. Errors for fitting parameters shown in parentheses.

	Cyclohexane	Methanol
Fraction of photoexcited molecules ^1^	20.0 (± 1.1)%	-
Fraction of direct, nonradiative relaxation back to the ground state ^2^	14.8 (± 0.9)%	-
Fraction of C_2_F_4_I· dissociating to C_2_F_4_ + I·	30 (± 1.1)%	20 (± 1.3)%
C_2_F_4_I· → C_2_F_4_ + I·	3.4 (± 2.9) × 10^9^ s^–1^	3.3 (± 2.1) × 10^9^ s^–1^
I· + I· → I_2_	1.1 (± 0.8) × 10^10^ M^–1^s^–1^	4.4 (± 1.3) × 10^10^ M^–1^s^–1^

^1^ Fraction of photoexcited molecules in 60 mM solution of ground-state C_2_F_4_I_2_. ^2^ Fraction of photoexcited molecules that relax back to the ground state without undergoing photodissociation.

**Table 2:** Key structural parameters of chemical species from global-fitting analysis in comparison with those from DFT calculations performed with various functionals.

Species	Structural Parameters ^1^	Optimized Values ^2^	ωB97X/def2-TZVPP	M06-2X/def2-TZVPP	B3LYP-D3/def2-TZVPP
*anti*-C_2_F_4_I_2_	r(C−I)	2.145 (± 0.03) Å	2.137 Å	2.147 Å	2.203 Å
	∠C−C−I	112.4 (± 9.8)°	111.7°	111.1°	111.4°
*gauche*-C_2_F_4_I_2_	r(C−I)	2.140 (± 0.05) Å	2.137 Å	2.138 Å	2.188 Å
	∠C−C−I	115.6 (± 10.4)°	113.5°	113.5°	114.0°
*anti*-C_2_F_4_I·	r(C−I)	2.156 (± 0.04) Å	2.163 Å	2.176 Å	2.248 Å
	∠C−C−I	119.2 (± 8.2)°	111.8°	111.8°	112.2°
*gauche*-C_2_F_4_I·	r(C−I)	2.138 (± 0.05) Å	2.129 Å	2.137 Å	2.191 Å
	∠C−C−I	118.1 (± 9.4)°	111.0°	111.0°	110.1°
I_2_	r(I−I)	2.661(± 0.08) Å	2.657 Å	2.653 Å	2.702 Å

^1^ Parameters refer to the structural parameters of each chemical species. Parameters consisting of two atomic symbols represent bond lengths, and parameters consisting of three atomic symbols represent bond angles. ^2^ Errors for global-fitting parameters shown in parentheses. Other parameters not shown here are fixed at obtained values from the DFT calculations (ωB97X/def2-TZVPP) shown in Table S2.

**Table 3:** Root-mean-square deviation (RMSD) values for DFT structures calculated with various combinations of functionals and basis sets. RMSD values are with respect to the experimental structural parameters determined from GFA of TRXL data. Among tested functionals, the ωB97X functional had the best agreement with the experimental values, regardless of basis set.

	ωB97X	M06-2X	B3LYP-D3	PBE0	TPSSh
def2-TZVPP	0.007 Å	0.010 Å	0.065 Å	0.032 Å	0.036 Å
	4.4°	4.4°	4.5°	5.8°	5.8°
cc-pVTZ(-PP) ^1^	0.009 Å	0.014 Å	0.075 Å	0.069 Å	0.045 Å
	4.5°	4.5°	4.7°	5.7°	5.6°
aug-cc-pVTZ(-PP) ^2^	0.007 Å	0.013 Å	0.071 Å	0.038 Å	0.041 Å
	4.4°	4.4°	4.5°	5.6°	5.6°

^1^ cc-pVTZ-PP small-core relativistic core potential (RECP) was used for iodine, and cc-pVTZ basis sets were used for other atoms. ^2^ aug-cc-pVTZ-PP small-core relativistic core potential (RECP) was used for iodine, and aug-cc-pVTZ all-electron basis sets were used for other atoms.

## Supplementary Materials

The following are available online at https://www.mdpi.com/article/10.3390/ijms22189793/s1.

## References

[1] Mulliken R.S. (1940). Intensities in Molecular Electronic Spectra X. Calculations on Mixed-Halogen, Hydrogen Halide, Alkyl Halide, and Hydroxyl Spectra. J. Chem. Phys..

[2] Kim J., Jun S., Kim J., Ihee H. (2009). Density Functional and ab Initio Investigation of CF_2_ICF_2_I and CF_2_CF_2_I Radicals in Gas and Solution Phases. J. Phys. Chem. A.

[3] Kalume A., George L., Cunningham N., Reid S.A. (2013). Case of the Missing Isomer: Pathways for Molecular Elimination in the Photoinduced Decomposition of 1,1-Dibromoethane. J. Phys. Chem. A.

[4] Kwok W.M., Ma C., Phillips D., Parker A.W., Towrie M., Matousek P., Phillips D.L. (2001). Picosecond time-resolved resonance Raman observation of Iso-CH_2_Br–I following A-band photodissociation of CH _2_BrI in the solution phase. Chem. Phys. Lett..

[5] Lee J.H., Kim J., Cammarata M., Kong Q., Kim K.H., Choi J., Kim T.K., Wulff M., Ihee H. (2008). Transient X-ray Diffraction Reveals Global and Major Reaction Pathways for the Photolysis of Iodoform in Solution. Angew. Chem. Int. Ed..

[6] Davidsson J., Poulsen J., Cammarata M., Georgiou P., Wouts R., Katona G., Jacobson F., Plech A., Wulff M., Nyman G. (2005). Structural Determination of a Transient Isomer of CH_2_I_2_ by Picosecond X-Ray Diffraction. Phys. Rev. Lett..

[7] Kong Q., Wulff M., Lee J.H., Bratos S., Ihee H. (2007). Photochemical Reaction Pathways of Carbon Tetrabromide in Solution Probed by Picosecond X-ray Diffraction. J. Am. Chem. Soc..

[8] Kong Q., Kim J., Lorenc M., Kim T.K., Ihee H., Wulff M. (2005). Photodissociation Reaction of 1,2-Diiodoethane in Solution:  A Theoretical and X-ray Diffraction Study. J. Phys. Chem. A.

[9] Lee J.H., Kim T.K., Kim J., Kong Q., Cammarata M., Lorenc M., Wulff M., Ihee H. (2008). Capturing Transient Structures in the Elimination Reaction of Haloalkane in Solution by Transient X-ray Diffraction. J. Am. Chem. Soc..

[10] Ihee H., Lorenc M., Kim T.K., Kong Q.Y., Cammarata M., Lee J.H., Bratos S., Wulff M. (2005). Ultrafast X-ray Diffraction of Transient Molecular Structures in Solution. Science.

[11] Ihee H. (2009). Visualizing solution-phase reaction dynamics with time-resolved X-ray liquidography. Acc. Chem. Res..

[12] Kim J., Lee J.H., Kim J., Jun S., Kim K.H., Kim T.W., Wulff M., Ihee H. (2012). Structural Dynamics of 1,2-Diiodoethane in Cyclohexane Probed by Picosecond X-ray Liquidography. J. Phys. Chem. A.

[13] Vincent J., Andersson M., Eklund M., Wöhri A.B., Odelius M., Malmerberg E., Kong Q., Wulff M., Neutze R., Davidsson J. (2009). Solvent dependent structural perturbations of chemical reaction intermediates visualized by time-resolved x-ray diffraction. J. Chem. Phys..

[14] Marcellini M., Nasedkin A., Zietz B., Petersson J., Vincent J., Palazzetti F., Malmerberg E., Kong Q., Wulff M., van der Spoel D. (2018). Transient isomers in the photodissociation of bromoiodomethane. J. Chem. Phys..

[15] Ahn C.W., Ki H., Kim J., Kim J., Park S., Lee Y., Kim K.H., Kong Q., Moon J., Pedersen M.N. (2018). Direct observation of a transiently formed isomer during iodoform photolysis in solution by time-resolved X-ray liquidography. J. Phys. Chem. Lett..

[16] Park S., Choi J., Ki H., Kim K.H., Oang K.Y., Roh H., Kim J., Nozawa S., Sato T., Adachi S.-i. (2019). Fate of transient isomer of CH_2_I_2_: Mechanism and origin of ionic photoproducts formation unveiled by time-resolved x-ray liquidography. J. Chem. Phys..

[17] Gerck E. (1983). Quantum yields of I (^2^P_1/2_) for CF_3_I, C_2_F_5_I, *i*-C_3_F_7_I, *n*-C_3_F_7_I, *n*-C_6_F_13_I, and 1, 2-C_2_F_4_I_2_ at 308 and 248 nm. J. Chem. Phys..

[18] Thomassen H., Samdal S., Hedberg K. (1992). Conformational analysis. 15. 1, 2-Dibromotetrafluoroethane and 1, 2-diiodotetrafluoroethane. Electron diffraction investigations of the molecular structures, compositions, and anti-gauche energy and entropy differences. J. Am. Chem. Soc..

[19] Minton T.K. (1986). Photofragmentation Dynamics of Iodohaloethanes. Ph.D. Thesis.

[20] Khundkar L.R., Zewail A.H. (1990). Picosecond photofragment spectroscopy. IV. Dynamics of consecutive bond breakage in the reaction C_2_F_4_I_2_→ C_2_F_4_+ 2I. J. Chem. Phys..

[21] Ihee H., Goodson B.M., Srinivasan R., Lobastov V.A., Zewail A.H. (2002). Ultrafast Electron Diffraction and Structural Dynamics: Transient Intermediates in the Elimination Reaction of C_2_F_4_I_2_. J. Phys. Chem. A.

[22] Reckenthaeler P., Centurion M., Fuß W., Trushin S.A., Krausz F., Fill E.E. (2009). Time-Resolved Electron Diffraction from Selectively Aligned Molecules. Phys. Rev. Lett..

[23] Wilkin K.J., Parrish R.M., Yang J., Wolf T.J., Nunes J.P.F., Guehr M., Li R., Shen X., Zheng Q., Wang X. (2019). Diffractive imaging of dissociation and ground-state dynamics in a complex molecule. Phys. Rev. A.

[24] Ihee H., Lobastov V.A., Gomez U.M., Goodson B.M., Srinivasan R., Ruan C.-Y., Zewail A.H. (2001). Direct Imaging of Transient Molecular Structures with Ultrafast Diffraction. Science.

[25] Ihee H., Kua J., Goddard W.A., Zewail A.H. (2001). CF_2_XCF_2_X and CF_2_XCF_2_• Radicals (X = Cl, Br, I):  Ab Initio and DFT Studies and Comparison with Experiments. J. Phys. Chem. A.

[26] Rasmusson M., Tarnovsky A.N., Pascher T., Sundström V., Åkesson E. (2002). Photodissociation of CH_2_ICH_2_I, CF_2_ICF_2_I, and CF_2_BrCF_2_I in solution. J. Phys. Chem. A.

[27] Park S., Lee T., Shin J., Yoon H., Pak Y., Lim M. (2020). Conformer-Specific Photodissociation Dynamics of CF_2_ICF_2_I in Solution Probed by Time-Resolved Infrared Spectroscopy. J. Phys. Chem. B.

[28] Tarnovsky A.N., Sundström V., Åkesson E., Pascher T. (2004). Photochemistry of Diiodomethane in Solution Studied by Femtosecond and Nanosecond Laser Photolysis. Formation and Dark Reactions of the CH_2_I−I Isomer Photoproduct and Its Role in Cyclopropanation of Olefins. J. Phys. Chem. A.

[29] Zheng X., Kwok W.M., Phillips D.L. (2000). Solvation Effects on the A-Band Photodissociation of Dibromomethane:  Turning a Photodissociation into a Photoisomerization. J. Phys. Chem. A.

[30] Kong Q., Khakhulin D., Shkrob I.A., Lee J.H., Zhang X., Kim J., Kim K.H., Jo J., Kim J., Kang J. (2019). Solvent-dependent complex reaction pathways of bromoform revealed by time-resolved X-ray solution scattering and X-ray transient absorption spectroscopy. Struct. Dyn..

[31] Haupa K.A., Lim M., Lee Y.-P. (2018). Photodissociation of CF_2_ICF_2_I in solid *para*-hydrogen: Infrared spectra of *anti*- and *gauche*-˙C_2_F_4_I radicals. Phys. Chem. Chem. Phys..

[32] Roeterdink W.G., Rijs A.M., Janssen M.H.M. (2006). Imaging of Ultrafast Molecular Elimination Reactions. J. Am. Chem. Soc..

[33] Chen Y.-H., Cheng Y.-K., Lee Y.-P. (2021). Formation and Infrared Spectrum of the Open-Form 2-Bromoethyl Radical (2-C_2_H_4_Br^•^) from Ultraviolet Irradiation of a C_2_H_4_/Br_2_/*p*-H_2_ Matrix. J. Phys. Chem. A.

[34] Brum J.L., Deshmukh S., Wang Z., Koplitz B. (1993). Site-specific branching ratios for H-atom production from primary haloalkanes photolyzed at 193, 222, and 248 nm. J. Chem. Phys..

[35] Kwok W.M., Fuss W., Phillips D.L. (1996). Bond selective electronic excitation in IC_2_H_4_C_2_F_4_I investigated by resonance Raman spectroscopy. Chem. Phys. Lett..

[36] Man S.Q., Kwok W.M., Phillips D.L., Johnson A.E. (1996). Short-time photodissociation dynamics of A-band and B-band bromoiodomethane in solution: An examination of bond selective electronic excitation. J. Chem. Phys..

[37] Zheng X., Phillips D.L. (1998). A-band resonance Raman spectra and short-time photodissociation dynamics of trans 1-chloro-2-iodoethane in cyclohexane solution. Chem. Phys. Lett..

[38] Panman M.R., Biasin E., Berntsson O., Hermann M., Niebling S., Hughes A.J., Kübel J., Atkovska K., Gustavsson E., Nimmrich A. (2020). Observing the Structural Evolution in the Photodissociation of Diiodomethane with Femtosecond Solution X-Ray Scattering. Phys. Rev. Lett..

[39] Park S., Shin J., Yoon H., Pak Y., Lim M. (2019). Complete photodissociation dynamics of CF_2_I_2_ in solution. Phys. Chem. Chem. Phys..

[40] Kim H., Kim J.G., Kim T.W., Lee S.J., Nozawa S., Adachi S.-I., Yoon K., Kim J., Ihee H. (2021). Ultrafast structural dynamics of in-cage isomerization of diiodomethane in solution. Chem. Sci..

[41] Butler L., Hintsa E., Shane S.F., Lee Y.-T. (1987). The Electronic State-selective Photodissociation of CH_2_BrI at 248, 210, and 193 nm. J. Chem. Phys..

[42] Lin J.J., Chen Y., Lee Y.Y., Lee Y.T., Yang X. (2002). Photodissociation dynamics of CH_3_Cl at 157.6 nm: Evidence for CH_2_(X^~3^B_1_/a^~1^A_1_)+HCl product channels. Chem. Phys. Lett..

[43] Odelius M., Kadi M., Davidsson J., Tarnovsky A.N. (2004). Photodissociation of diiodomethane in acetonitrile solution and fragment recombination into iso-diiodomethane studied with *ab* initio molecular dynamics simulations. J. Chem. Phys..

[44] Park S., Shin J., Yoon H., Lim M. (2020). Photodissociation Dynamics of CF_2_I_2_ in CCl_4_ Solution Probed by Femtosecond Infrared Spectroscopy. Bull. Korean Chem. Soc..

[45] Cox A.P., Duxbury G., Hardy J.A., Kawashima Y. (1980). Microwave spectra of CF_3_Br and CF_3_I. Structures and dipole moments. J. Chem. Soc. Faraday Trans. 2.

[46] Engels B., Peyerimhoff S.D. (1986). Theoretical study of the bridging in β-halo ethyl. J. Mol. Struct..

[47] Zhong D., Ahmad S., Zewail A. (1997). Femtosecond Elimination Reaction Dynamics. J. Am. Chem. Soc..

[48] Ihee H., Zewail A.H., Goddard W.A. (1999). Conformations and barriers of haloethyl radicals (CH_2_XCH_2_, X= F, Cl, Br, I): Ab initio studies. J. Phys. Chem. A.

[49] Skell P.S., Tuleen D.L., Readio P.D. (1963). Stereochemical Evidence of Bridged Radicals. J. Am. Chem. Soc..

[50] Skell P.S., Traynham J.G. (1984). Radical brominations of alkyl bromides and the nature of *β*-bromoalkyl radicals. Acc. Chem. Res..

[51] Kim K.H., Kim J., Lee J.H., Ihee H. (2014). Topical Review: Molecular reaction and solvation visualized by time-resolved X-ray solution scattering: Structure, dynamics, and their solvent dependence. Struct. Dyn..

[52] Kim T.K., Lee J.H., Wulff M., Kong Q., Ihee H. (2009). Spatiotemporal Kinetics in Solution Studied by Time-Resolved X-Ray Liquidography (Solution Scattering). ChemPhysChem.

[53] Kim J., Kim K.H., Oang K.Y., Lee J.H., Hong K., Cho H., Huse N., Schoenlein R.W., Kim T.K., Ihee H. (2016). Tracking reaction dynamics in solution by pump–probe X-ray absorption spectroscopy and X-ray liquidography (solution scattering). Chem. Commun..

[54] Lee J.H., Wulff M., Bratos S., Petersen J., Guerin L., Leicknam J.-C., Cammarata M., Kong Q., Kim J., Møller K.B. (2013). Filming the Birth of Molecules and Accompanying Solvent Rearrangement. J. Am. Chem. Soc..

[55] Kim T.K., Lorenc M., Lee J.H., Russo M.L., Kim J., Cammarata M., Kong Q., Noel S., Plech A., Wulff M. (2006). Spatiotemporal reaction kinetics of an ultrafast photoreaction pathway visualized by time-resolved liquid x-ray diffraction. Proc. Natl. Acad. Sci. USA.

[56] Leshchev D., Khakhulin D., Newby G., Ki H., Ihee H., Wulff M. (2019). Sub-nanosecond secondary geminate recombination in mercury halides HgX_2_ (X = I, Br) investigated by time-resolved x-ray scattering. J. Chem. Phys..

[57] Choi E.H., Ahn D.-S., Park S., Kim C., Ahn C.W., Kim S., Choi M., Yang C., Kim T.W., Ki H. (2019). Structural Dynamics of Bismuth Triiodide in Solution Triggered by Photoinduced Ligand-to-Metal Charge Transfer. J. Phys. Chem. Lett..

[58] Kong Q., Lee J.H., Kim K.H., Kim J., Wulff M., Ihee H., Koch M.H. (2010). Ultrafast X-ray solution scattering reveals different reaction pathways in the photolysis of triruthenium dodecacarbonyl (Ru_3_(CO)_12_) after ultraviolet and visible excitation. J. Am. Chem. Soc..

[59] Biasin E., van Driel T.B., Kjær K.S., Dohn A.O., Christensen M., Harlang T., Vester P., Chabera P., Liu Y., Uhlig J. (2016). Femtosecond X-Ray Scattering Study of Ultrafast Photoinduced Structural Dynamics in Solvated [Co(terpy)_2_]^2+^. Phys. Rev. Lett..

[60] Christensen M., Haldrup K., Bechgaard K., Feidenhans’l R., Kong Q., Cammarata M., Russo M.L., Wulff M., Harrit N., Nielsen M.M. (2009). Time-Resolved X-ray Scattering of an Electronically Excited State in Solution. Structure of the ^3^A_2u_ State of Tetrakis-μ-pyrophosphitodiplatinate(II). J. Am. Chem. Soc..

[61] Kim K.H., Oang K.Y., Kim J., Lee J.H., Kim Y., Ihee H. (2011). Direct observation of myoglobin structural dynamics from 100 picoseconds to 1 microsecond with picosecond X-ray solution scattering. Chem. Commun..

[62] Kim T.W., Lee J.H., Choi J., Kim K.H., van Wilderen L.J., Guerin L., Kim Y., Jung Y.O., Yang C., Kim J. (2012). Protein Structural Dynamics of Photoactive Yellow Protein in Solution Revealed by Pump–Probe X-ray Solution Scattering. J. Am. Chem. Soc..

[63] Kim J.G., Kim T.W., Kim J., Ihee H. (2015). Protein Structural Dynamics Revealed by Time-Resolved X-ray Solution Scattering. Acc. Chem. Res..

[64] Yang C., Choi M., Kim J.G., Kim H., Muniyappan S., Nozawa S., Adachi S.-i., Henning R., Kosheleva I., Ihee H. (2018). Protein Structural Dynamics of Wild-Type and Mutant Homodimeric Hemoglobin Studied by Time-Resolved X-Ray Solution Scattering. Int. J. Mol. Sci..

[65] Choi M., Kim J.G., Muniyappan S., Kim H., Kim T.W., Lee Y., Lee S.J., Kim S.O., Ihee H. (2021). Effect of the abolition of intersubunit salt bridges on allosteric protein structural dynamics. Chem. Sci..

[66] Cho H.S., Dashdorj N., Schotte F., Graber T., Henning R., Anfinrud P. (2010). Protein structural dynamics in solution unveiled via 100-ps time-resolved x-ray scattering. Proc. Natl. Acad. Sci. USA.

[67] Lee S.J., Kim Y., Kim T.W., Yang C., Thamilselvan K., Jeong H., Hyun J., Ihee H. (2021). Reversible molecular motional switch based on circular photoactive protein oligomers exhibits unexpected photo-induced contraction. Cell. Rep. Phys. Sci.

[68] Kim K.H., Kim J.G., Oang K.Y., Kim T.W., Ki H., Jo J., Kim J., Sato T., Nozawa S., Adachi S.-I. (2016). Femtosecond X-ray solution scattering reveals that bond formation mechanism of a gold trimer complex is independent of excitation wavelength. Struct. Dyn..

[69] Choi E.H., Kim J.G., Kim J., Ki H., Lee Y., Lee S., Yoon K., Kim J., Kim J., Ihee H. (2021). Filming ultrafast roaming-mediated isomerization of bismuth triiodide in solution. Nat. Commun..

[70] Lee Y., Kim J.G., Lee S.J., Muniyappan S., Kim T.W., Ki H., Kim H., Jo J., Yun S.R., Lee H. (2021). Ultrafast coherent motion and helix rearrangement of homodimeric hemoglobin visualized with femtosecond X-ray solution scattering. Nat. Commun..

[71] Kim J.G., Nozawa S., Kim H., Choi E.H., Sato T., Kim T.W., Kim K.H., Ki H., Kim J., Choi M. (2020). Mapping the emergence of molecular vibrations mediating bond formation. Nature.

[72] Kim K.H., Ki H., Lee J.H., Park S., Kong Q., Kim J., Kim J., Wulff M., Ihee H. (2015). Solvent-dependent structure of molecular iodine probed by picosecond X-ray solution scattering. Phys. Chem. Chem. Phys..

[73] Wulff M., Bratos S., Plech A., Vuilleumier R., Mirloup F., Lorenc M., Kong Q., Ihee H. (2006). Recombination of photodissociated iodine: A time-resolved x-ray-diffraction study. J. Chem. Phys..

[74] Jun S., Lee J.H., Kim J., Kim J., Kim K.H., Kong Q., Kim T.K., Lo Russo M., Wulff M., Ihee H. (2010). Photochemistry of HgBr_2_ in methanol investigated using time-resolved X-ray liquidography. Phys. Chem. Chem. Phys..

[75] Kim K.H., Lee J.H., Kim J., Nozawa S., Sato T., Tomita A., Ichiyanagi K., Ki H., Kim J., Adachi S.-i. (2013). Solvent-Dependent Molecular Structure of Ionic Species Directly Measured by Ultrafast X-Ray Solution Scattering. Phys. Rev. Lett..

[76] Dribinski V., Barbera J., Martin J.P., Svendsen A., Thompson M.A., Parson R., Lineberger W.C. (2006). Time-resolved study of solvent-induced recombination in photodissociated IBr^−^(CO_2_)_n_ clusters. J. Chem. Phys..

[77] Murov S. Properties of Solvents Used in Organic Chemistry. http://murov.info/orgsolvents.htm#TABLE%202.

[78] Lomont J.P., Shearer A.J., Nguyen S.C., Harris C.B. (2013). Picosecond TRIR Studies of M_3_(CO)_12_ (M = Fe, Os) Clusters in Solution. Organometallics.

[79] Lampert R.A., Chewter L.A., Phillips D., O’Connor D.V., Roberts A.J., Meech S.R. (1983). Standards for nanosecond fluorescence decay time measurements. Anal. Chem..

[80] Dawson W.R., Windsor M.W. (1968). Fluorescence yields of aromatic compounds. J. Phys. Chem..

[81] Weigel W., Rettig W., Dekhtyar M., Modrakowski C., Beinhoff M., Schlüter A.D. (2003). Dual Fluorescence of Phenyl and Biphenyl Substituted Pyrene Derivatives. J. Phys. Chem. A.

[82] Nozawa S., Adachi S.I., Takahashi J.I., Tazaki R., Guérin L., Daimon M., Tomita A., Sato T., Chollet M., Collet E. (2007). Developing 100 ps-resolved X-ray structural analysis capabilities on beamline NW14A at the Photon Factory Advanced Ring. J. Synchrotron Rad..

[83] Ichiyanagi K., Sato T., Nozawa S., Kim K.-H., Lee J.-H., Choi J., Tomita A., Ichikawa H., Adachi S., Ihee H. (2009). 100 ps time-resolved solution scattering utilizing a wide-bandwidth X-ray beam from multilayer optics. J. Synchrotron Rad..

[84] James F., Roos M. (1975). Minuit-a system for function minimization and analysis of the parameter errors and correlations. Comput. Phys. Commun..

[85] Kim J., Kim T.K., Ihee H. (2011). Density Functional and Spin− Orbit Ab Initio Study of CF_3_Br: Molecular Properties and Electronic Curve Crossing. J. Phys. Chem. A.

[86] Shao Y., Gan Z., Epifanovsky E., Gilbert A.T., Wormit M., Kussmann J., Lange A.W., Behn A., Deng J., Feng X. (2015). Advances in molecular quantum chemistry contained in the Q-Chem 4 program package. Mol. Phys..

[87] Barone V., Cossi M. (1998). Quantum Calculation of Molecular Energies and Energy Gradients in Solution by a Conductor Solvent Model. J. Phys. Chem. A.

[88] Cossi M., Rega N., Scalmani G., Barone V. (2003). Energies, structures, and electronic properties of molecules in solution with the C-PCM solvation model. J. Comput. Chem..

[89] Zhao Y., Truhlar D.G. (2008). The M06 suite of density functionals for main group thermochemistry, thermochemical kinetics, noncovalent interactions, excited states, and transition elements: Two new functionals and systematic testing of four M06-class functionals and 12 other functionals. Theor. Chem. Acc..

[90] Beck A.D. (1993). Density-functional thermochemistry. III. The role of exact exchange. J. Chem. Phys.

[91] Stephens P.J., Devlin F.J., Chabalowski C.F., Frisch M.J. (1994). Ab Initio Calculation of Vibrational Absorption and Circular Dichroism Spectra Using Density Functional Force Fields. J. Phys. Chem..

[92] Grimme S., Antony J., Ehrlich S., Krieg H. (2010). A consistent and accurate ab initio parametrization of density functional dispersion correction (DFT-D) for the 94 elements H-Pu. J. Chem. Phys..

[93] Adamo C., Barone V. (1999). Toward reliable density functional methods without adjustable parameters: The PBE0 model. J. Chem. Phys..

[94] Staroverov V.N., Scuseria G.E., Tao J., Perdew J.P. (2003). Comparative assessment of a new nonempirical density functional: Molecules and hydrogen-bonded complexes. J. Chem. Phys..

[95] Pritchard B.P., Altarawy D., Didier B., Gibson T.D., Windus T.L. (2019). New Basis Set Exchange: An Open, Up-to-Date Resource for the Molecular Sciences Community. J. Chem. Inf. Model..

[96] Kennedy I., Geering H., Rose M., Crossan A. (2019). A simple method to estimate entropy and free energy of atmospheric gases from their action. Entropy.

