# Supplementary material for: Structural Dynamics of C2F4I2 in Cyclohexane Studied via Time-Resolved X-ray Liquidography
Source: Int J Mol Sci. 2021 Sep 10;22(18):9793. doi: 10.3390/ijms22189793 (PMC8469616; doi:10.3390/ijms22189793)
Supplement: Supplementary file 1 [file ijms-22-09793-s001.zip › C2F4I2 SI_revised_Ihee.pdf]

Supporting Information for

Structural Dynamics of $C_2F_4I_2$ in Cyclohexane Studied via Time-Resolved X-ray Liquidography

Jain Gu[1,2,†], Seonggon Lee[1,2,†], Seunghwan Eom[1,2], Hosung Ki[1,2], Eun Hyuk Choi[1,2], Yunbeom Lee[1,2], Shunsuke Nozawa[3,4], Shin-ichi Adachi[3,4], Jeongho Kim[5], Hyotcherl Ihee[1,2,*]

[*]To whom correspondence should be addressed. E-mail: hyotcherl.ihee@kaist.ac.kr

This PDF file includes:

Supporting Methods S1-S2

Supporting Figures S1-S5

Supporting Tables S1-S2

Supporting References

Supporting Methods

Method S1. Experimental artifacts

In general, a difference scattering curve at a negative time delay should be flat for the following reason. The difference scattering curve at a negative time delay (for example, −200 ps) is obtained as follows.

$$\Delta S(q, t = -200 \text{ ps}) = S(q, t = -200 \text{ ps}) - S(q, t = -2 \text{ ns}) \quad \text{(S1)}$$

This equation indicates that the difference scattering curve, $q\Delta S(q, t = -200$ ps), contains the difference of signal intensities at $t = -200$ ps and $t = -2$ ns. Since both $t = -200$ ps and $t = -2$ ns precede time zero, that is, the moment of laser excitation, both $qS(q, t = -200$ ps) and $qS(q, t = -2$ ns) are measured from the same reactant solution without any reaction initiated, and therefore two curves should be identical. In other words, $\Delta S(q, t = -200$ ps) is supposed to be flat in an ideal case. Nevertheless, as shown in Figure S3, the difference curve at a negative time delay is not flat for the data shown in this work. This observation indicates that an artifact, whose exact origin is not clear, exists in the experimental data. We assumed that the signal at the negative time delay directly represents the shape of the artifact. More specifically, we determined the shape of the experimental artifact, $\Delta S(q)_{\text{artifact}}$, in q-space by using the following equation.

$$\Delta S(q)_{\text{artifact}} = \Delta S(q, t = -200 \text{ ps}) \quad \text{(S2)}$$

Accordingly, we removed this contribution of the experimental artifacts from $\Delta S(q, t)$ to extract more accurate structural and kinetic information from the data. The contribution of the experimental artifact was simply removed as follows.

$$\Delta S(q, t)_{\text{art\_rem}} = \Delta S(q, t)_{\text{raw\_exp}} - \Delta S(q, t = -200 \text{ ps}) \quad \text{(S3)}$$

Here, $\Delta S(q, t)_{\text{raw\_exp}}$ and $\Delta S(q, t)_{\text{art\_rem}}$ are the experimental data before and after removing the contribution of the experimental artifact, respectively. The time-resolved difference scattering

curves shown in Figures 1 and 4 are $q\Delta S(q, t)_{art\_rem}$, and difference RDFs shown in Figures 1, 2, and 4 are obtained by the sine-Fourier transform of $q\Delta S(q, t)_{art\_rem}$ curves.

Method S2. Hydrodynamic response of solvent

First of all, the hydrodynamic response was obtained via a separate solvent heating experiment, where 4-bromo-4'-(*N*,*N*-diethylamino)-azobenzene (CAS number: 22700-62-5) dissolved in cyclohexane with 7.18 mM concentration was used. The TRXL data from the dye solution was then analyzed to extract the hydrodynamic response of cyclohexane solvent [1,2]. Unlike TRXL data in other solvents such as methanol and water, which do not suffer from laser-induced evaporation, the TRXL data in cyclohexane often suffer from unusual artifact caused by laser-induced evaporation at a high laser fluence [3]. Such artifacts can be avoided if the data are collected at a very low laser fluence, but in this mode, the signal-to-noise ratio becomes too low. Therefore, we used a normal laser fluence and later removed the artifact from the laser-induced solvent evaporation. For this purpose, we performed the solvent heating experiment of the dye solution at two laser powers (0.21 and 1.19 mJ/mm^2) (see Figure S4(a)). When a high fluence (1.19 mJ/mm^2) of laser excitation is used, it was found that the amplitude of $\Delta S(q)$ increases significantly at t = 1 μs, especially at low q values (< 3.5 Å^{-1}). The anomalous increase of $\Delta S(q)$ at the low q region is attributed to rapid vaporization of cyclohexane [3]. Considering such a power dependency of the signal, we expressed the hydrodynamics response of solvent as a linear combination of three terms, $(\partial S/\partial T)_\rho$, $(\partial S/\partial \rho)_T$, and an additional component $\Delta S(q)_{vap}$, which is calculated as follows.

$$\Delta S(q)_{vap} = \Delta S(q, t = 1\ \mu s)_{high\_power} - \Delta S(q, t = 1\ \mu s)_{low\_power} \quad (S4)$$

Here, $\Delta S(q, t = 1\ \mu s)_{high\_power}$ and $\Delta S(q, t = 1\ \mu s)_{low\_power}$ denote the difference scattering curves of the dye solution measured with two different laser powers, 1.19 and 0.21 mJ/mm^2, respectively, at t = 1 μs. The contribution of the $\Delta S(q)_{vap}$ component was used as an additional term to the theoretical $\Delta S(q, t)$ in the GFA. Figure S4(b) shows the contribution of $\Delta S(q)_{vap}$ at

all time delays obtained from GFA. It can be seen that the magnitude of $\Delta S(q)_{vap}$ is largest at 1 μs.

Supporting Figures

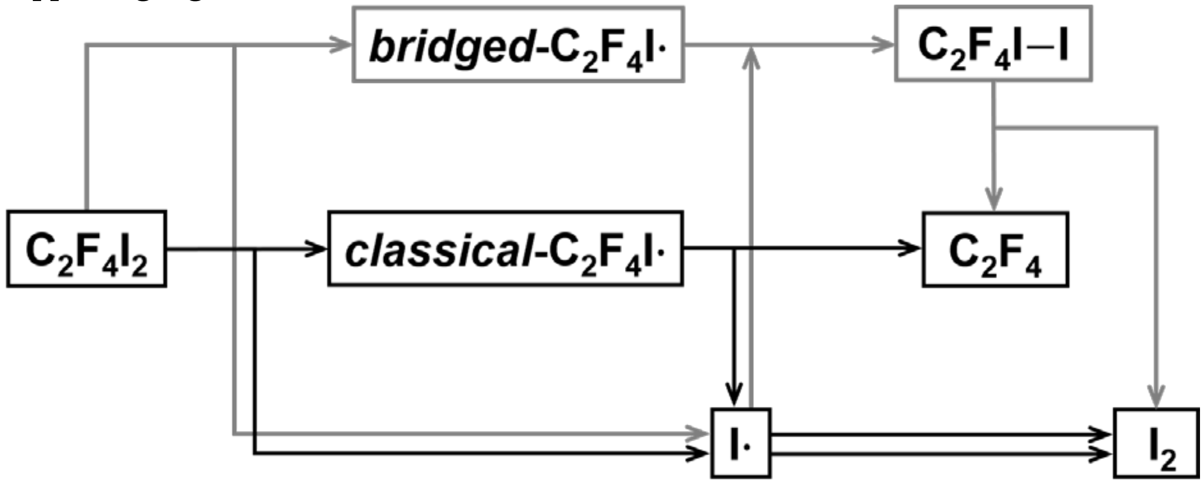

Figure S1. Kinetic scheme considering all possible intermediates and products in the reaction. Black lines stand for reaction pathways of *classical* forms (*anti* and *gauche*) of the $C_2F_4I\cdot$ radical, and gray lines stand for reaction pathways involving the *bridged* form of the $C_2F_4I\cdot$ radical.

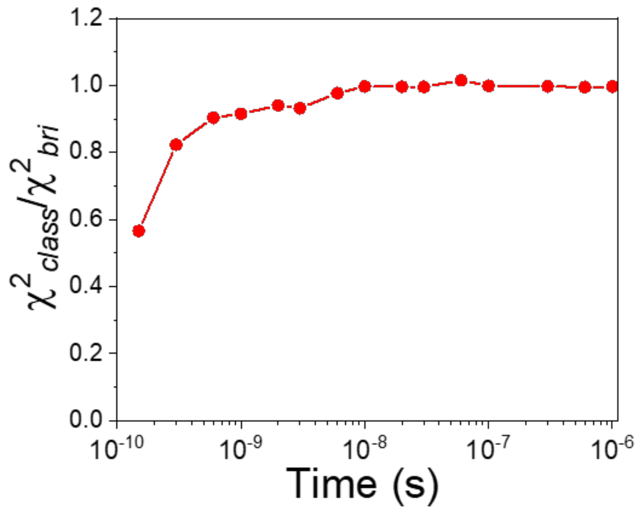

Figure S2. The ratio of the reduced chi-square values between the *classical* and *bridged* models, $\chi^2_{class}/\chi^2_{bri}$. At 150 ps, where the concentration of $C_2F_4I\cdot$ is high, the ratio is significantly smaller than 1, indicating that the *bridged* model fits the experimental data much better than the *classical* model. As the concentration of $C_2F_4I\cdot$ decreases at later time delays, the ratio expectedly approaches 1.

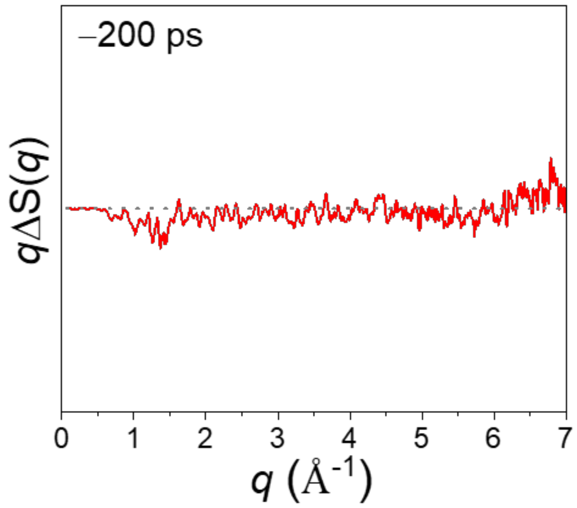

Figure. S3. The $q\Delta S(q, t)$ curve at a negative time delay ($t = -200$ ps).

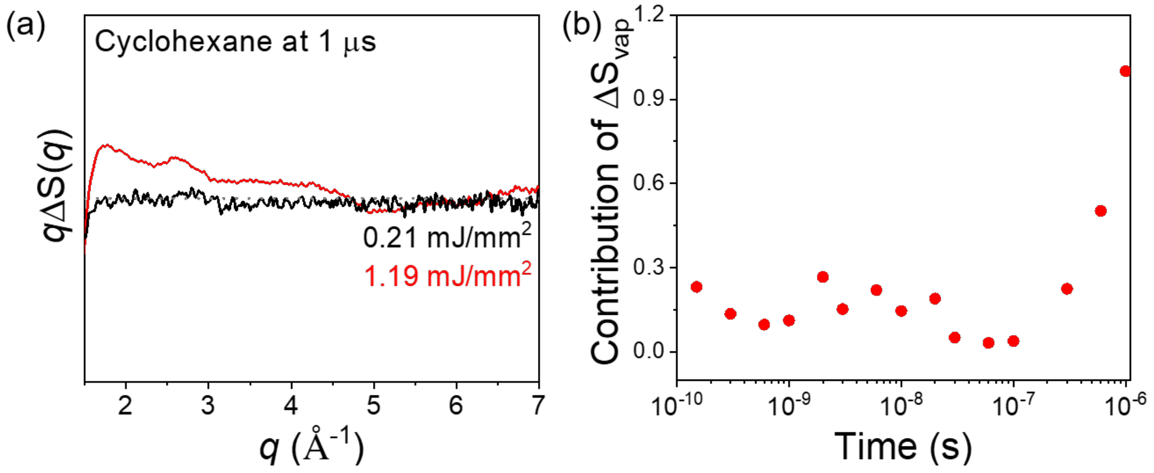

Figure S4. Artifact scattering, $\Delta S(q)_{vap}$, arising from the response of cyclohexane solvent under a strong laser fluence. (a) Difference scattering curves of the dye solution in cyclohexane at two laser powers (0.21 and 1.19 mJ/mm^2). The amplitude of the difference scattering curve with a high laser fluence (1.19 mJ/mm^2, red line) increases significantly at 1 μs whereas such increase is absent with a low laser fluence (0.21 mJ/mm^2, black line). (b) Contribution of $\Delta S(q)_{vap}$ at each time delay obtained from GFA.

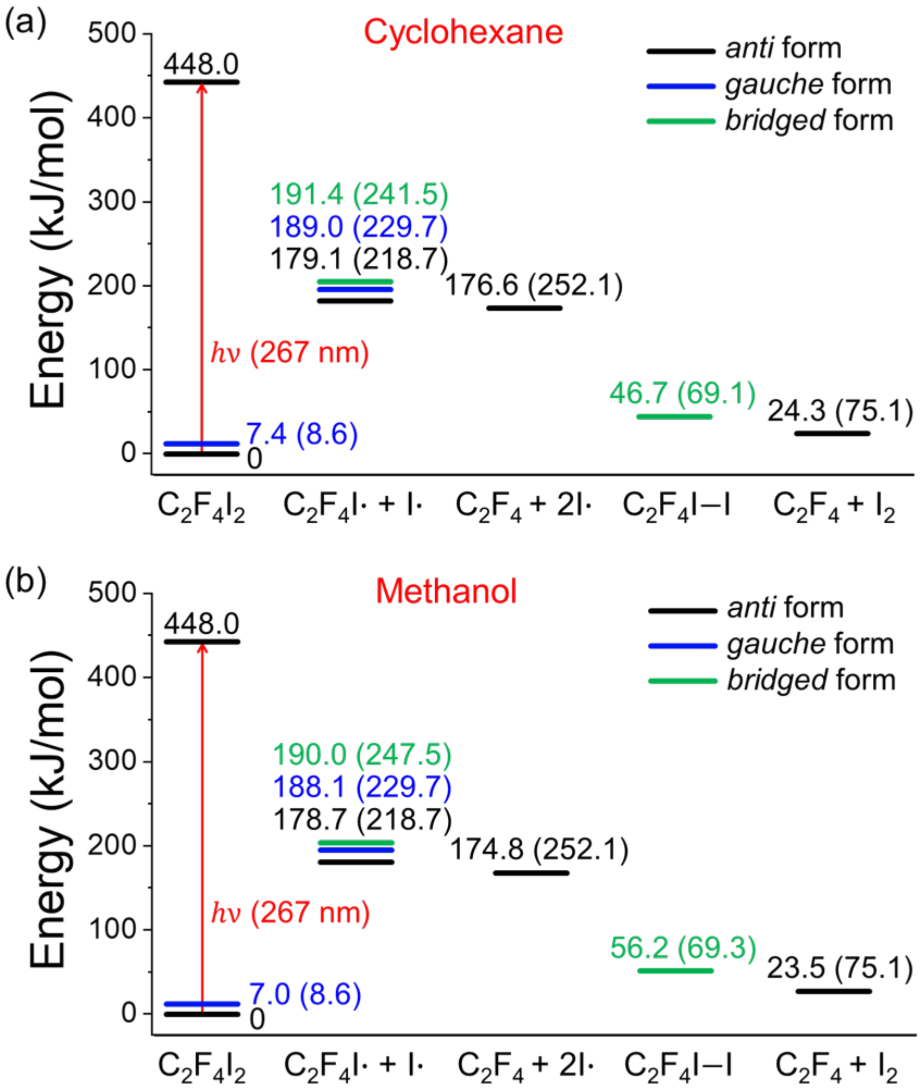

Figure S5. Energy diagram for the reaction channels starting from $C_2F_4I_2$ in (a) cyclohexane and (b) methanol. The energy values were calculated by the DFT method using the ωB97X functional and the def2-TZVPP basis set. The energies (kJ/mol) shown in the diagram are relative to the energy of the *anti*-$C_2F_4I_2$ parent molecule in the ground state in each solvent. The values are Gibbs free energies and those in parentheses are enthalpies.

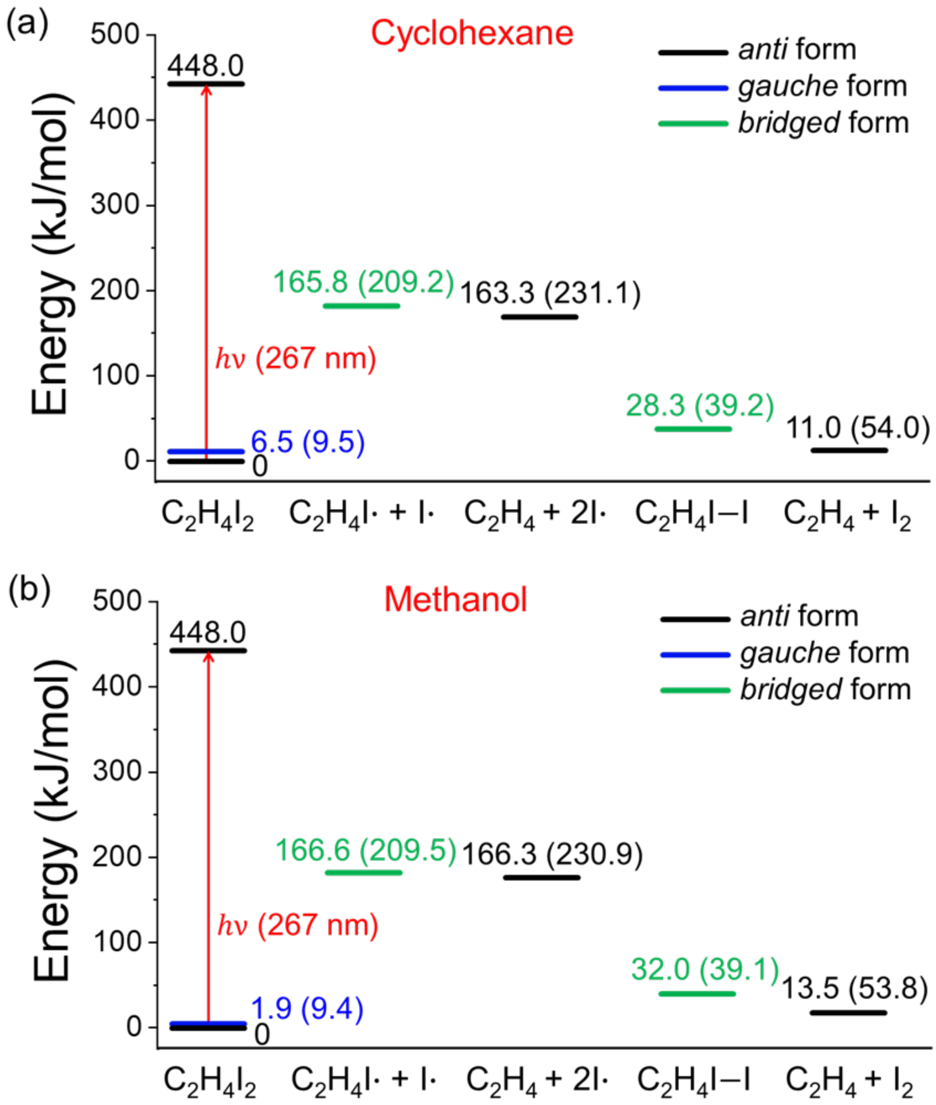

Figure S6. Energy diagram for the reaction channels starting from $C_2H_4I_2$ in (a) cyclohexane and (b) methanol. The energy values were calculated by the DFT method using the ωB97X functional and the def2-TZVPP basis set. The energies (kJ/mol) shown in the diagram are relative to the energy of the *anti*-$C_2H_4I_2$ parent molecule in the ground state in each solvent. The values are Gibbs free energies and those in parentheses are enthalpies.

Supporting Table

Table S1. Bimolecular rate constants of I_2 formation, ΔT, and Δ*ρ* in various solvents, including the data reported in previous TRXL studies.

**Solvent**	**Solute**	**Bimolecular rate constants of I_2 formation ($\times 10^{10}$ $M^{-1}s^{-1}$)**	**ΔT (K) [1]**	**Δ*ρ* (kg/m^3) [2]**	**Ref.**
Cyclohexane	$C_2H_4I_2$	1.58 ± 0.10	3.4 (10 ns)	−2.5	[4]
	CHI_3	1.52 ± 0.01	1.7 (-) [3]	- [4]	[3]
	CH_2I_2	0.65	- [4]	- [4]	[5]
	$C_2F_4I_2$	1.1 ± 0.8	1.15 (10 ns)	−0.86	This work
Methanol	$C_2H_4I_2$	- [4]	2.48 (10 ns)	−2.5	[6]
	CHI_3	3.1 ± 0.5	1.15 (10 ns)	−1.2	[7]
	$C_2F_4I_2$	4.4 ± 1.3	- [4]	- [4]	[8]
	HgI_2	3.3 ± 0.5	0.76 (10 ns)	−0.82	[9]
Acetonitrile	BiI_3	1.90 ± 0.48	0.12 (100 ns)	−0.95	[10]
	HgI_2	0.6 ± 0.2	0.4 (10 ns)	−0.38	[11]

[1] The maximum ΔT is shown, and the corresponding time delay is shown in parenthesis.
[2] The value at 1 μs is shown.
[3] The time profile of ΔT is not reported. Instead, the value at equilibrium is shown.
[4] Not available.

Table S2. Structural parameters obtained from the structures optimized by DFT calculation in the gas phase, methanol, and cyclohexane. The parameters were optimized with the scalar relativistic effect being considered by the introduction of ωB97X/def2-TZVPP small-core relativistic effective core potential (RECP) on the iodine atom.

Species	Parameters [2]	In gas	In methanol	In cyclohexane [1]
anti-$C_2F_4I_2$	*r*(C−C)	1.551 Å	1.551 Å	1.551 Å
	r(C−F)	1.339 Å	1.341 Å	1.340 Å
	r(C−I)	2.137 Å	2.136 Å	2.137 Å
	r(I···I)	5.055 Å	5.058 Å	5.057 Å
	∠C−C−F	108.9°	108.7°	108.9°
	∠F−C−F	108.2°	108.0°	108.1°
	∠C−C−I	111.6°	111.9°	111.7°
	∠F−C−I	109.6°	109.7°	109.7°
	∠I−C−C−I	180°	180°	180°
gauche-$C_2F_4I_2$	*r*(C−C)	1.558 Å	1.557 Å	1.558 Å
	r(C−F)	1.341 Å	1.343 Å	1.340 Å
	r(C−I)	2.137 Å	2.135 Å	2.137 Å
	r(I···I)	3.877 Å	3.887 Å	3.883 Å

	∠C−C−F	107.4°	107.9°	108.5°
	∠F−C−F	107.8°	107.4°	107.6°
	∠C−C−I	113.3°	113.6°	113.5°
	∠F−C−I	109.8°	109.9°	109.8°
	∠I−C−C−I	65.1°	65.1°	65.2°
anti-C_2F_4I [3]	$r(C_a{-}C_b)$	1.504 Å	1.505 Å	1.505 Å
	$r(C_a{-}F)$	1.338 Å	1.340 Å	1.339 Å
	$r(C_b{-}F)$	1.314 Å	1.315 Å	1.315 Å
	$r(C_a{-}I)$	2.164 Å	2.162 Å	2.163 Å
	∠$C_a{-}C_b{-}F$	115.2°	115.2°	115.2°
	∠$C_b{-}C_a{-}F$	109.1°	109.1°	109.2°
	∠$F{-}C_a{-}F$	108.8°	108.6°	108.7°
	∠$F{-}C_b{-}F$	112.0°	112.0°	112.0°
	∠$C_b{-}C_a{-}I$	111.7°	112.0°	111.8°
	∠$F{-}C_a{-}I$	109.0°	109.1°	109.0°
gauche-C_2F_4I [3]	$r(C_a{-}C_b)$	1.514 Å	1.513 Å	1.514 Å

	r(C$_a$−F)	1.319 Å	1.350 Å	1.341 Å
	r(C$_b$−F)	1.319 Å	1.319 Å	1.320 Å
	r(C$_a$−I)	2.130 Å	2.128 Å	2.129 Å
	∠C$_a$−C$_b$−F	114.0°	114.1°	114.7°
	∠C$_b$−C$_a$−F	109.0°	109.2°	109.2°
	∠F−C$_a$−F	107.2°	106.9°	107.1°
	∠F−C$_b$−F	111.4°	111.4°	111.4°
	∠C$_b$−C$_a$−I	111.9°	112.2°	111.0°
	∠F−C$_a$−I	109.7°	110.2°	110.1°
bridged-C_2F_4I	r(C−C)	1.325 Å	1.526 Å	1.325 Å
	r(C−F)	1.310 Å	1.328 Å	1.311 Å
	r(C−I)	3.408 Å	2.349 Å	3.402 Å
	∠C−C−F	123.2°	115.6°	123.2°
	∠F−C−F	113.5°	110.1°	113.5°
	∠C−C−I	78.8°	71.1°	78.8°
	∠F−C−I	97.3°	119.7°	97.4°

bridged-C_2F_4I−I	*r*(C−C)	1.320 Å	1.318 Å	1.319 Å
	r(C−F)	1.313 Å	1.314 Å	1.314 Å
	r(C−I)	3.730 Å	3.803 Å	3.491 Å
	r(I−I)	2.657 Å	2.660 Å	2.658 Å
	∠C−C−F	123.3°	123.4°	123.4°
	∠F−C−F	113.3°	113.2°	113.3°
	∠C−C−I	79.7°	80.0°	79.9°
	∠F−C−I	96.2°	96.1°	96.2°
C_2F_4	*r*(C−C)	1.317 Å	1.316 Å	1.317 Å
	r(C−F)	1.315 Å	1.315 Å	1.315 Å
	∠C−C−F	123.4°	123.5°	123.4°
	∠F−C−F	113.2°	113.1°	113.1°
I_2	*r*(I−I)	2.655 Å	2.660 Å	2.657 Å

[1] These values were used in the GFA of the TRXL data. The structural parameters shown in Table 2 were optimized by the fitting whereas the other parameters were fixed at the values shown here.

[2] The parameters refer to the structural parameters of each chemical species. The parameters consisting of two atomic symbols represent bond lengths and the parameters consisting of three atomic symbols represent bond angles. The parameters consisting of four atomic symbols represent dihedral angles.

[3] The carbon atom bonded with iodine is labeled as C_a and the other carbon atom as C_b.

Table S3. Structural parameters obtained from the structures optimized by DFT calculation with various combinations of five functionals (ωB97X, M06-2X, B3LYP-D3, PBE0, and TPSSh) and three basis sets (def2-TZVPP, cc-pVTZ(PP), and aug-cc-pVTZ(PP)) in cyclohexane. The three values in each block represent the values calculated using the basis set of def2-TZVPP, cc-pVTZ(PP), and aug-cc-pVTZ(PP) in the appearing order.

Species	Parameters [1]	ωB97X	M06-2X	B3LYP-D3	PBE0	TPSSh
anti-$C_2F_4I_2$	*r*(C−C)	1.551 Å	1.542 Å	1.552 Å	1.544 Å	1.553 Å
		1.548 Å	1.539 Å	1.548 Å	1.542 Å	1.550 Å
		1.551 Å	1.541 Å	1.552 Å	1.545 Å	1.553 Å
	r(C−F)	1.340 Å	1.332 Å	1.341 Å	1.331 Å	1.343 Å
		1.338 Å	1.332 Å	1.338 Å	1.329 Å	1.341 Å
		1.340 Å	1.333 Å	1.340 Å	1.331 Å	1.343 Å
	r(C−I)	2.137 Å	2.147 Å	2.203 Å	2.165 Å	2.169 Å
		2.145 Å	2.152 Å	2.214 Å	2.176 Å	2.178 Å
		2.143 Å	2.151 Å	2.210 Å	2.173 Å	2.175 Å
	r(I···I)	5.057 Å	5.057 Å	5.175 Å	5.087 Å	5.099 Å
		5.066 Å	5.062 Å	5.191 Å	5.099 Å	5.109 Å
		5.068 Å	5.065 Å	5.188 Å	5.100 Å	5.109 Å
	∠C−C−F	108.9°	109.3°	109.7°	109.6°	109.3°
		108.9°	109.2°	109.7°	109.7°	109.4°
		108.9°	109.1°	109.6°	109.7°	109.4°
	∠F−C−F	108.1°	108.4°	108.7°	108.7°	108.3°

		108.3°	108.5°	108.8°	108.9°	108.5°
		108.1°	108.4°	108.6°	108.7°	108.3°
	∠C−C−I	111.7°	111.1°	111.4°	110.8°	110.8°
		111.5°	111.0°	111.1°	110.6°	110.5°
		111.7°	111.1°	111.4°	110.6°	110.7°
	∠F−C−I	109.7°	109.6°	108.8°	109.1°	109.6°
		109.6°	109.5°	108.7°	109.0°	109.5°
		109.6°	109.5°	108.8°	109.0°	109.6°
	∠I−C−C−I	180.0°	180.0°	175.8°	180.0°	179.9°
		180.0°	180.0°	179.7°	175.1°	180.0°
		180.0°	180.0°	175.3°	180.0°	180.0°
gauche-$C_2F_4I_2$	*r*(C−C)	1.558 Å	1.552 Å	1.565 Å	1.554 Å	1.562 Å
		1.555 Å	1.549 Å	1.563 Å	1.552 Å	1.560 Å
		1.556 Å	1.551 Å	1.565 Å	1.554 Å	1.562 Å
	r(C−F)	1.340 Å	1.335 Å	1.344 Å	1.334 Å	1.345 Å
		1.340 Å	1.335 Å	1.342 Å	1.333 Å	1.344 Å
		1.340 Å	1.337 Å	1.344 Å	1.334 Å	1.346 Å
	r(C−I)	2.137 Å	2.138 Å	2.188 Å	2.155 Å	2.165 Å
		2.144 Å	2.143 Å	2.196 Å	2.163 Å	2.173 Å
		2.143 Å	2.143 Å	2.195 Å	2.162 Å	2.168 Å
	r(I···I)	3.883 Å	3.876 Å	4.057 Å	3.920 Å	3.838 Å

		3.893 Å	3.887 Å	4.073 Å	3.940 Å	3.862 Å
		3.893 Å	3.887 Å	4.068 Å	3.933 Å	3.845 Å
	∠C−C−F	108.5°	107.9°	108.2°	107.4°	108.5°
		108.0°	107.9°	108.3°	108.5°	108.5°
		108.0°	107.9°	108.3°	108.5°	108.5°
	∠F−C−F	107.6°	107.7°	107.8°	108.0°	107.7°
		107.8°	107.8°	107.9°	108.1°	107.9°
		107.6°	107.7°	107.8°	107.9°	107.7°
	∠C−C−I	113.5°	113.5°	114.0°	112.9°	112.2°
		113.4°	113.7°	113.9°	112.8°	112.2°
		113.5°	113.6°	113.9°	112.8°	112.2°
	∠F−C−I	109.8°	109.8°	109.2°	109.5°	109.9°
		109.7°	109.7°	109.1°	109.4°	109.8°
		109.8°	109.8°	109.2°	109.5°	109.9°
	∠I−C−C−I	65.2°	64.6°	70.2°	68.0°	63.9°
		65.6°	65.1°	71.0°	68.9°	64.4°
		65.3°	64.7°	70.5°	68.4°	64.0°
anti-C_2F_4I [2]	$r(C_a{-}C_b)$	1.505 Å	1.497 Å	1.491 Å	1.489 Å	1.498 Å
		1.502 Å	1.494 Å	1.488 Å	1.486 Å	1.495 Å
		1.505 Å	1.496 Å	1.492 Å	1.489 Å	1.498 Å
	$r(C_a{-}F)$	1.339 Å	1.333 Å	1.341 Å	1.331 Å	1.342 Å

	1.337 Å	1.332 Å	1.338 Å	1.329 Å	1.340 Å
	1.339 Å	1.334 Å	1.341 Å	1.331 Å	1.342 Å
$r(C_b\text{–}F)$	1.315 Å	1.309 Å	1.315 Å	1.307 Å	1.320 Å
	1.314 Å	1.310 Å	1.315 Å	1.307 Å	1.320 Å
	1.315 Å	1.310 Å	1.316 Å	1.307 Å	1.320 Å
$r(C_a\text{–}I)$	2.163 Å	2.176 Å	2.248 Å	2.211 Å	2.216 Å
	2.173 Å	2.182 Å	2.260 Å	2.222 Å	2.228 Å
	2.170 Å	2.180 Å	2.254 Å	2.217 Å	2.222 Å
$\angle C_a\text{–}C_b\text{–}F$	115.2°	115.4°	116.5°	116.3°	115.9°
	115.1°	115.2°	116.4°	116.3°	115.9°
	115.2°	115.3°	116.5°	116.3°	115.9°
$\angle C_b\text{–}C_a\text{–}F$	109.2°	109.5°	110.1°	110.0°	109.8°
	109.3°	109.6°	110.4°	110.2°	110.1°
	109.2°	109.5°	110.2°	110.1°	109.9°
$\angle F\text{–}C_a\text{–}F$	108.7°	109.0°	109.3°	109.4°	109.0°
	108.9°	109.1°	109.5°	109.6°	109.2°
	108.7°	108.9°	109.3°	109.4°	109.0°
$\angle F\text{–}C_b\text{–}F$	112.0°	112.3°	112.5°	112.6°	112.1°
	112.1°	112.3°	112.6°	112.7°	112.2°
	112.0°	112.2°	112.5°	112.7°	112.0°
$\angle C_b\text{–}C_a\text{–}I$	111.8°	111.8°	112.2°	112.1°	111.3°

		111.5°	111.5°	111.7°	111.7°	111.1°
		111.8°	111.7°	112.1°	112.6°	111.3°
	∠ F−C_a−I	109.0°	108.6°	107.6°	107.7°	109.0°
		108.8°	108.5°	107.4°	107.5°	108.2°
		109.0°	108.6°	107.5°	107.7°	108.3°
gauche-C_2F_4I [2]	*r*(C_a−C_b)	1.514 Å	1.511 Å	1.511 Å	1.507 Å	1.514 Å
		1.512 Å	1.501 Å	1.510 Å	1.505 Å	1.523 Å
		1.514 Å	1.501 Å	1.512 Å	1.507 Å	1.514 Å
	r(C_a−F)	1.341 Å	1.340 Å	1.349 Å	1.339 Å	1.352 Å
		1.346 Å	1.340 Å	1.347 Å	1.338 Å	1.351 Å
		1.347 Å	1.341 Å	1.350 Å	1.339Å	1.353 Å
	r(C_b−F)	1.320 Å	1.315 Å	1.324 Å	1.315 Å	1.327 Å
		1.320 Å	1.316 Å	1.324 Å	1.315 Å	1.327 Å
		1.319 Å	1.316 Å	1.324 Å	1.315 Å	1.327 Å
	r(C_a−I)	2.129 Å	2.137 Å	2.191 Å	2.157 Å	2.158 Å
		2.137 Å	2.142 Å	2.200 Å	2.166 Å	2.166 Å
		2.136 Å	2.141 Å	2.197 Å	2.163 Å	2.163 Å
	∠ C_a−C_b−F	114.7°	114.1°	115.0°	115.0°	114.9°
		114.6°	113.9°	114.9°	114.8°	114.8°
		114.7°	114.1°	115.0°	115.0°	114.9°
	∠ C_b−C_a−F	109.2°	109.3°	110.1°	110.2°	109.8°

		109.3°	109.3°	110.3°	110.4°	110.0°
		109.3°	109.3°	110.2°	110.3°	109.9°
	∠F−C_a−F	107.1°	107.4°	107.6°	107.7°	107.1°
		107.2°	107.5°	107.8°	107.9°	107.2°
		107.1°	107.4°	107.6°	107.7°	107.1°
	∠F−C_b−F	111.4°	111.4°	111.7°	111.7°	111.1°
		111.5°	111.4°	111.7°	111.8°	111.2°
		111.4°	111.3°	111.6°	111.6°	111.1°
	∠C_b−C_a−I	111.0°	111.0°	110.1°	109.6°	109.9°
		111.1°	111.0°	110.0°	109.4°	109.8°
		111.0°	111.1°	110.2°	109.6°	109.9°
	∠F−C_a−I	110.1°	109.9°	109.4°	109.5°	110.1°
		110.0°	109.8°	109.2°	109.4°	109.9°
		110.1°	109.9°	109.3°	109.5°	110.1°
bridged-C_2F_4I	*r*(C−C)	1.325 Å	1.323 Å	1.344 Å	1.346 Å	1.351 Å
		1.326 Å	1.322 Å	1.344 Å	1.346 Å	1.351 Å
		1.325 Å	1.322 Å	1.342 Å	1.346 Å	1.351 Å
	r(C−F)	1.311 Å	1.308 Å	1.307 Å	1.297 Å	1.305 Å
		1.310 Å	1.308 Å	1.307 Å	1.297 Å	1.305 Å
		1.311 Å	1.308 Å	1.338 Å	1.297 Å	1.306 Å
	r(C−I)	3.402 Å	3.303 Å	3.144 Å	3.040 Å	3.064 Å

		3.342 Å	3.268 Å	3.140 Å	3.035 Å	3.052 Å
		3.397 Å	3.293 Å	3.150 Å	3.043 Å	3.064 Å
	∠C−C−F	123.2°	123.2°	122.8°	122.7°	122.6°
		123.2°	123.2°	122.8°	122.6°	122.6°
		123.2°	123.2°	122.0°	122.7°	122.6°
	∠F−C−F	113.5°	113.6°	114.2°	114.4°	114.4°
		113.6°	113.7°	114.3°	114.5°	114.5°
		113.5°	113.6°	115.9°	114.4°	114.4°
	∠C−C−I	78.8°	78.5°	77.7°	77.2°	77.3°
		78.6°	78.3°	77.6°	77.2°	77.2°
		78.6°	78.4°	77.8°	77.2°	77.3°
	∠F−C−I	97.4°	97.0°	99.0°	99.5°	99.6°
		97.5°	96.9°	98.9°	99.4°	99.6°
		97.3°	96.9°	98.3°	99.4°	99.6°
bridged-C_2F_4I−I	*r*(C−C)	1.319 Å	1.321 Å	1.326 Å	1.325 Å	1.325 Å
		1.319 Å	1.319 Å	1.326 Å	1.347 Å	1.322 Å
		1.319 Å	1.319 Å	1.326 Å	1.325 Å	1.319 Å
	r(C−F)	1.314 Å	1.309 Å	1.317 Å	1.308 Å	1.320 Å
		1.314 Å	1.314 Å	1.317 Å	1.313 Å	1.317 Å
		1.314 Å	1.314 Å	1.318 Å	1.309 Å	1.321 Å
	r(C−I)	3.491 Å	3.344 Å	3.555 Å	3.505 Å	4.037 Å

		3.691 Å	3.691 Å	3.490 Å	3.316 Å	3.304 Å
		3.799 Å	3.799 Å	3.566 Å	3.538 Å	3.756 Å
	r(I−I)	2.658 Å	2.660 Å	2.705 Å	2.668 Å	2.676 Å
		2.664 Å	2.664 Å	2.712 Å	2.708 Å	2.700 Å
		2.666 Å	2.666 Å	2.712 Å	2.676 Å	2.720 Å
	∠C−C−F	123.4°	123.3°	123.3°	123.3°	123.4°
		123.3°	123.3°	123.2°	123.6°	123.2°
		123.4°	123.4°	123.3°	123.3°	123.6°
	∠F−C−F	113.3°	113.5°	113.4°	113.5°	113.2°
		113.3°	113.3°	113.5°	112.8°	113.6°
		113.2°	113.2°	113.4°	113.4°	112.7°
	∠C−C−I	79.9°	78.6°	79.3°	79.1°	80.6°
		79.7°	79.7°	79.1°	79.8°	78.4°
		80.0°	80.0°	79.3°	79.2°	79.9°
	∠F−C−I	96.2°	96.7°	96.5°	96.8°	95.9°
		96.2°	96.2°	96.5°	97.4°	97.5°
		96.2°	96.2°	96.6°	96.7°	97.1°
C_2F_4	*r*(C−C)	1.317 Å	1.316 Å	1.321 Å	1.320 Å	1.323 Å
		1.316 Å	1.315 Å	1.321 Å	1.319 Å	1.323 Å
		1.317 Å	1.315 Å	1.321 Å	1.320 Å	1.323 Å
	r(C−F)	1.315 Å	1.311 Å	1.320 Å	1.311 Å	1.321 Å

		1.315 Å	1.312 Å	1.320 Å	1.311 Å	1.321 Å
		1.315 Å	1.312 Å	1.320 Å	1.311 Å	1.321 Å
	∠C−C−F	123.4°	123.4°	123.4°	123.4°	123.5°
		123.4°	123.4°	123.4°	123.4°	123.4°
		123.5°	123.4°	123.4°	123.4°	123.5°
	∠F−C−F	113.1°	113.2°	113.2°	113.2°	113.1°
		113.2°	113.3°	113.2°	113.3°	113.2°
		113.1°	113.2°	113.1°	113.2°	113.1°
I_2	r(I−I)	2.657 Å	2.653 Å	2.702 Å	2.665 Å	2.674 Å
		2.664 Å	2.659 Å	2.709 Å	2.671 Å	2.682 Å
		2.665 Å	2.660 Å	2.709 Å	2.672 Å	2.682 Å

[1] The parameters refer to the structural parameters of each chemical species. The parameters consisting of two atomic symbols represent bond lengths and the parameters consisting of three atomic symbols represent bond angles. The parameters consisting of four atomic symbols represent dihedral angles.

[2] The carbon atom bonded with iodine is labeled as C_a and the other carbon atom as C_b.

Supporting References

1. Panman, M.R.; Biasin, E.; Berntsson, O.; Hermann, M.; Niebling, S.; Hughes, A.J.; Kübel, J.; Atkovska, K.; Gustavsson, E.; Nimmrich, A. Observing the Structural Evolution in the Photodissociation of Diiodomethane with Femtosecond Solution X-Ray Scattering. *Phys. Rev. Lett.* **2020**, *125*, 226001, doi:10.1103/PhysRevLett.125.226001.
2. Kjær, K.S.; van Driel, T.B.; Kehres, J.; Haldrup, K.; Khakhulin, D.; Bechgaard, K.; Cammarata, M.; Wulff, M.; Sørensen, T.J.; Nielsen, M.M. Introducing a standard method for experimental determination of the solvent response in laser pump, X-ray probe time-resolved wide-angle X-ray scattering experiments on systems in solution. *Phys. Chem. Chem. Phys.* **2013**, *15*, 15003-15016, doi:10.1039/C3CP50751C
3. Ahn, C.W.; Ki, H.; Kim, J.; Kim, J.; Park, S.; Lee, Y.; Kim, K.H.; Kong, Q.; Moon, J.; Pedersen, M.N. Direct observation of a transiently formed isomer during iodoform photolysis in solution by time-resolved X-ray liquidography. *J. Phys. Chem. Lett.* **2018**, *9*, 647-653, doi:10.1021/acs.jpclett.7b03125.
4. Kim, J.; Lee, J.H.; Kim, J.; Jun, S.; Kim, K.H.; Kim, T.W.; Wulff, M.; Ihee, H. Structural Dynamics of 1,2-Diiodoethane in Cyclohexane Probed by Picosecond X-ray Liquidography. *J. Phys. Chem. A* **2012**, *116*, 2713-2722, doi:10.1021/jp2078314.
5. Vincent, J.; Andersson, M.; Eklund, M.; Wöhri, A.B.; Odelius, M.; Malmerberg, E.; Kong, Q.; Wulff, M.; Neutze, R.; Davidsson, J. Solvent dependent structural perturbations of chemical reaction intermediates visualized by time-resolved x-ray diffraction. *J. Chem. Phys.* **2009**, *130*, 154502, doi:10.1063/1.3111401.
6. Cammarata, M.; Lorenc, M.; Kim, T.; Lee, J.-H.; Kong, Q.; Pontecorvo, E.; Lo Russo, M.; Schiro, G.; Cupane, A.; Wulff, M. Impulsive solvent heating probed by picosecond x-ray diffraction. *J. Chem. Phys.* **2006**, *124,* 124504, doi:10.1063/1.2176617.

7. Lee, J.H.; Kim, J.; Cammarata, M.; Kong, Q.; Kim, K.H.; Choi, J.; Kim, T.K.; Wulff, M.; Ihee, H. Transient X-ray Diffraction Reveals Global and Major Reaction Pathways for the Photolysis of Iodoform in Solution. *Angew. Chem. Int. Ed.* **2008**, *120,* 1063-1066, doi:10.1002/anie.200704150.

8. Lee, J.H.; Kim, T.K.; Kim, J.; Kong, Q.; Cammarata, M.; Lorenc, M.; Wulff, M.; Ihee, H. Capturing Transient Structures in the Elimination Reaction of Haloalkane in Solution by Transient X-ray Diffraction. *J. Am. Chem. Soc.* **2008**, *130*, 5834-5835, doi:10.1021/ja710267u.

9. Kim, T.K.; Lorenc, M.; Lee, J.H.; Russo, M.L.; Kim, J.; Cammarata, M.; Kong, Q.; Noel, S.; Plech, A.; Wulff, M. Spatiotemporal reaction kinetics of an ultrafast photoreaction pathway visualized by time-resolved liquid x-ray diffraction. *Proc. Natl. Acad. Sci. USA* **2006**, *103*, 9410-9415, doi:10.1073/pnas.0601958103.

10. Choi, E.H.; Ahn, D.-S.; Park, S.; Kim, C.; Ahn, C.W.; Kim, S.; Choi, M.; Yang, C.; Kim, T.W.; Ki, H. Structural Dynamics of Bismuth Triiodide in Solution Triggered by Photoinduced Ligand-to-Metal Charge Transfer. *J. Phys. Chem. Lett.* **2019**, *10*, 1279-1285, doi:10.1021/acs.jpclett.9b00365.

11. Leshchev, D.; Khakhulin, D.; Newby, G.; Ki, H.; Ihee, H.; Wulff, M. Sub-nanosecond secondary geminate recombination in mercury halides HgX_2 (X= I, Br) investigated by time-resolved x-ray scattering. *J. Chem. Phys.* **2019**, *151*, 054310, doi:10.1063/1.5096422.
